# The effectiveness of time domain and nonlinear heart rate variability metrics in ultra‐short time series

**DOI:** 10.14814/phy2.15863

**Published:** 2023-11-27

**Authors:** Zifan Gu, Vanessa Zarubin, Carolyn Martsberger

**Affiliations:** ^1^ Department of Physics Wofford College Spartanburg South Carolina USA; ^2^ Department of Biomedical Informatics Harvard Medical School Boston Massachusetts USA; ^3^ Department of Psychology Northwestern University Evanston Illinois USA; ^4^ Present address: Quantitative Biomedical Research Center, Peter O'Donnell Jr. School of Public Health University of Texas Southwestern Medical Center Dallas Texas USA

**Keywords:** heart rate variability, International Affective Picture System (IAPS), stress, ultra‐short time series

## Abstract

Ultra short‐term (UST) heart rate variability (HRV) has been used to establish normative HRV values. This study aims to investigate whether HRV metrics can capture changes in HRV from external stimuli, and whether these metrics remain effective under various recording length. Participants completed varying stimulating activities including viewing images, arithmetic tasks, and memory recall of viewed images. SDNN, RMSSD, pNN50, SD2, SD1/SD2, and DFA were extracted from the data. Comparing arithmetic calculation and the first minute of memory recall, SDNN, pNN50, SD2, and SD1/SD2 had significant HRV differences; this suggests that these metrics can distinguish the inherently different stimuli participants were exposed to. However, comparing first minute of viewing with that of the second, SDNN, pNN50, and SD2, presented some significant HRV differences during two inherently similar stimuli. Comparing the first 60–120 s during viewing, SDNN, pNN50, and SD2 also presented significant differences. Our results suggest that SDNN, pNN50, and SD2 may not be robust in evaluating UST HRVs in replacement of the standard short‐term HRV. It may be beneficial to analyze multiple HRV metrics, particularly SD1/SD2, to achieve a more holistic understanding of the underlying physiology.

## 1 INTRODUCTION

Heart rate variability (HRV) quantifies the changes in successive heartbeats and is integral in psychophysiological research. Most commonly collected by electrocardiograms (ECG), the electrical signals from the heart show an association between the parasympathetic nervous system and cognitive mechanisms, affected by external stimuli (Electrophysiology TFotESoCatNASoPa, 1996). An increase in HRV augments mobility efficiency, whereas a decrease in HRV induces risk of mortality (Shaffer et al., 2020). Reduced HRV could be a marker for fetal distress (Hon & Lee, 1963), anxiety (Cohen & Benjamin, 2006), asthma (Kazuma et al., 1997), cardiac arrhythmias (Lehrer et al., 2004), chronic obstructive pulmonary diseases (Giardino et al., 2004), depression (Agelink et al., 2002), functional gastrointestinal disorders (Gevirtz, 2013), hypertension, inflammation, myocardial infarction (Berntson et al., 2008; Bigger Jr. et al., 1992; Carney et al., 2007), post‐traumatic stress disorder (PTSD; Shah et al., 2013), and sudden infant death (Hon & Lee, 1963). Increases in HRV correlate with resilience, adaptation to surroundings (Beckers et al., 2006), and strength in emotion regulations (Mather & Thayer, 2018). Exceptions to this trend include extreme cases in which disease increases the spatial and temporal complexity of HRV beyond what is exhibited in a healthy system and increases mortality risk (Shaffer & Ginsberg, 2017; Vaillancourt & Newell, 2002).

The minimally invasive but informative nature of ECG popularized HRV metrics and divides those metrics into three main categories: time domain, frequency domain, and nonlinear. In time‐domain metrics, methods include root mean square of successive RR interval differences (RMSSD), standard deviation of the normal‐normal intervals (SDNN), and the average difference of maximum HR and minimum HR (HR Max – HR Min; Shaffer & Ginsberg, 2017). In frequency domain metrics, methods include ultra‐low, very‐low, low, and high frequencies (ULF, VLF, LF, and HF, respectively). Nonlinear indices include the standard deviation of RR intervals on ellipses (SD1, SD2; Raetz et al., 1991; Tulppo et al., 1996) and detrended fluctuation analysis (DFA; Peng et al., 1995).

The duration of ECG recordings for computing HRV varies widely across studies, often motivated by study design and physiological features of interest, from long‐term (24 h) to short‐term (5 min) to ultra‐short‐term (UST); <5 min). The Task Force of the European Society of Cardiology and the North American Society of Pacing and Electrophysiology (Task Force) advises an optimal recording length of 5 min in order to make results across studies comparable, and that frequency domain metrics in those recordings are desired over time‐domain metrics (Electrophysiology TFotESoCatNASoPa, 1996). These recommendations are still widely used by the field, but since then, increasing number of studies have reported viability of UST HRV metrics using time‐domain metrics and nonlinear metrics. UST HRV metrics refer to metrics derived from recordings less than 5 min (Shaffer & Ginsberg, 2017). In the time‐domain metrics, examples include monitoring mental stress in mobile settings (Salahuddin et al., 2007) and the athletes during rest, exercise, and recovery (Esco & Flatt, 2014). Another study compares study between 10 and 300 s of ECG recordings (Munoz et al., 2015) to show potential of deriving HRV using SDNN, pNN50, and RMSSD from shorter segments. The Multi‐Ethnic Study of Atherosclerosis (MESA) also shows that ECG recordings as short as 10 s could effectively identify normative ranges of SDNN and RMSSD (O'Neal et al., 2016). In nonlinear metrics, university examinations and resting (Castaldo et al., 2019) and exercise with and without autonomic blockades (Tulppo et al., 1996) show similar potential using SD2 and the SD1/SD2 ratio. It is worth noting that fractal behavior, as demonstrated using DFA α1, have been reliably observed in RR intervals of 5 min or less (Gronwald et al., 2019; Heitmann et al., 2011). While these studies showed promise, Nussinovitch et al. analyzed both 10 s and 1‐min recordings of RR intervals and found that HRV triangular index, NN50, pNN50, SDNN, VLF, and HF power did not correlate well with their 5‐min counterparts (Nussinovitch et al., 2011).

Past studies report shifts of HRV can be induced by multiple types of stimuli, such as performing arithmetic tests and memory recalls (Gu et al., 2022), the Cold Pressor Stress Test (Ghiasi et al., 2018, 2020), and watching positive and negative videos (Barquero‐Perez et al., 2020; Ghiasi et al., 2020). This study focuses on how visual stimulation, task performance, and recording RR interval lengths impact HRV in UST through the metrics of RMSSD, SDNN, PNN50, SD2, and SD1/SD2 ratio. DFA has shown to be meaningful when used to quantify HRV under different mental tasks up to 1‐min intervals (Gu et al., 2022). The goal of this study is to investigate how well different HRV metrics can capture changes in the autonomic nervous system caused by external stimuli. The study will also assess the efficacy of UST‐HRV in the context of this study.

## 2 MATERIALS AND METHODS

Seventy‐three matriculated students (21 males) from Wofford College participated in this study. Participants were between 18 and 22 years old, who did not report any substance (drug or alcohol) abuse, who were not under general anesthesia 2 weeks prior to testing, and who did not experience traumatic physical event 30 days prior to testing. One participant with pulmonary embolism and two other participants with tachycardia were excluded from the study. Thirty‐four participants (12 males) were randomly assigned to the pure image regime, and 36 participants (nine males) were randomly assigned to the mixed image regime. The pure and mixed image regime is described below. All participants provided written informed consent, and procedures were approved by the Wofford College Institutional Review Board.

Each participant viewed nine lists of negative, categorical, or neutral images, determined by image ratings in arousal, valence, and relatedness. Arousal and valence ratings were determined in pilot studies where participants ranked each image on a 9‐point Likert scale from “calm/soothing” to “exciting/agitating” (arousal ratings) and from “very unpleasing” to “very pleasing” (valence ratings). Relatedness ratings were ranked on a 7‐point Likert scale from “low association” to “high association” (Zarubin et al., 2020). The images were selected from the International Affective Picture System (IAPS; Lang et al., 1997), the Geneva Affective Picture Database (Dan‐Glauser & Scherer, 2011), the Emotional Picture Set (Wessa et al., 2010), the image pool of Talmi et al. (2007), and Google Images. All participants saw the same pool of images; however, in the pure regime, each list consisted of only one type of image (three lists each of negative, categorical, and neutral images), and in the mixed regime, all nine lists consisted of a mix of the three image types.

**FIGURE 1:**
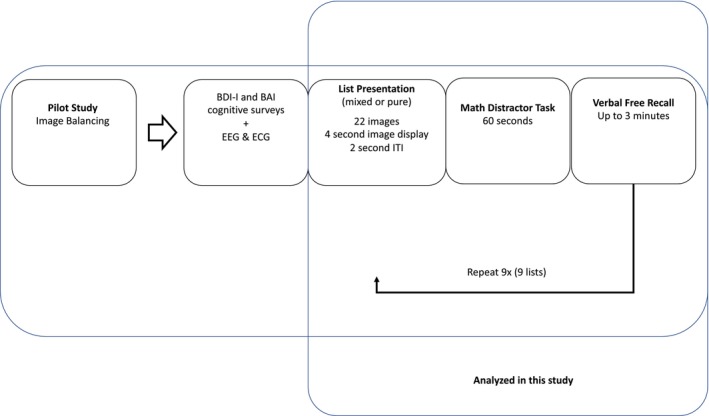
Timeline of participants during the study. Images were balanced during a pilot study. Participants completed cognitive and affective surveys while EEG and ECG nodes were attached to them. Participants were then presented with nine sets of images categorized as negative, categorical, or neutral images. All participants were exposed to the same pool of images. In the pure regime, each list exclusively comprised one type of image‐totaling three lists for each category. The mixed regime consisted of a blend of the three image types for each of the nine lists. At the end of each list, participants completed an arithmetic distractor test and then were asked to recall the images presented during the list.

At the start of the study, an ECG device was attached to participants before they completed the depression and anxiety questionnaires (BDI‐I and BAI) and a practice test, prior to the start of the study. Participants then viewed images in an upright seated position for 2 min and 18 s, where each image was active for 2 s followed by a 4 s fixation cross. After viewing 22 images, participants were asked to perform arithmetic calculations for 1 min and to perform memory recalls for up to 3 min. We used a variety of math problems in the distractor task, randomly selecting a different set of problems for each of the nine sessions. During memory recalls, participants reported verbally to research assistants on what they remember seeing in the previous viewing segment. The viewing of images is designated as “viewing” throughout this manuscript, whereas the arithmetic and memory recalls are designated as “tasks.” The viewing and task segments were repeated nine times, making the duration of this study approximately 1 h. The experimental timeline can be found in Figure 1. A 3‐lead ECG was acquired via 3 flat‐type electrodes (DA‐AT‐EXTOF1) attached to the EXG channels of the Cortech Active Two 32 Channel EEG system (Manufacturer: Cortech Solutions; Model Specifications: Model Number DA‐AT_HCL32) from the Behavioral Brain Sciences Center, Birmingham, United Kingdom. Data were sampled at 1.024 kHz, and Lead III was primarily used for analysis.

Out of the total time of 2 min and 18 s for viewing and up to 4 min for completing tasks in each segment, the first 2 min were used for analysis. The last 18 s in each segment were omitted to provide a uniform recording length for analysis, as well as to bring compatibility when comparing between tasks and viewing segments. It should be noted that during the first minute of completing tasks, participants were required to take an arithmetic test. Therefore, the 2 min of task analysis included a 1‐min arithmetic test and a 1‐min memory recall. Because participants were verbally reporting to research assistants on what they remembered and social evaluations may cause stress among participants (Schwabe et al., 2012), the analysis of these tasks required investigation at different resolutions (i.e., both at the 2‐min resolution and at the 1‐min resolution) to examine the predictive power of the six metrics. Lastly, to evaluate the sensitivity to length for these metrics, the first 60s and first 120 s of viewing were analyzed to assess UST viability. Participants who missed more than 5% of the recording were excluded from the segment of the analysis.

Time‐domain metrics SDNN, RMSSD, and pNN50, nonlinear metrics SD2, SD1/SD2 ratio, and DFA were analyzed for each time series. It should be noted that RMSSD and SD1 are identical metrics, both from a mathematical and an empirical perspective (Ciccone et al., 2017). Therefore, standalone SD1 is omitted from the results.

### 2.1 Time‐domain metrics

#### 2.1.1 Standard deviation of normal‐to‐normal

The standard deviation of normal‐to‐normal beats (SDNN) is the sample standard deviation given an array of time series. It is calculated as

$$\text{SDNN}=\sqrt{\frac{1}{N-1}\sum_{k=1}^{N}\;{\left\langle {\mathrm{RR}}_{k}-\overset{―}{\mathrm{RR}}\right\rangle }^{2}}$$

where *N* is the total number of RR intervals in the time series, $\overset{―}{\mathrm{RR}}$ is the arithmetic mean of those RR, and ${\mathrm{RR}}_{k}$ is the *k*th RR in the series. SDNN is viewed as the gold standard for recordings over 24 h (Electrophysiology TFotESoCatNASoPa, 1996; Shaffer & Ginsberg, 2017). Recently studies have shown effective SDNN derivation from recordings of 60s in mobile ECG (Salahuddin et al., 2007) and of 10s in standardized ECG (O'Neal et al., 2016).

#### 2.1.2 Root mean square of successive RR interval differences

The root mean square of successive RR interval differences (RMSSD) is the square root of the average squared differences between RR intervals. It can be calculated as

$$\text{RMSSD}=\sqrt{\frac{1}{N-1}\sum_{k=1}^{N-1}\;{\left\langle {\mathrm{RR}}_{k}-{\mathrm{RR}}_{k+1}\right\rangle }^{2}}$$

where *N* is the total number of RR intervals in the time series and ${\mathrm{RR}}_{k}$ is the *k*th RR in the series. RMSSD is a recommended metric for studies focusing on vagal tone (Laborde et al., 2017). It has also been shown that 30s ECG is sufficient in deriving RMSSD (Munoz et al., 2015).

#### 2.1.3 pNN50

Percentage of subsequent RR intervals differing more than 50 ms (pNN50) counts the number of RR matching the criteria and divides by the total number. It can be calculated as

$$\mathrm{pNN}50=\frac{{\mathrm{RR}}_{50}}{N-1}$$

where ${\mathrm{RR}}_{50}$ is the total number of RR intervals that differs more than 50 ms than its previous beat, and N is the total number of RR intervals. Baek et al. found that 60s ECG is sufficient to derive pNN50 (Baek et al., 2015).

### 2.2 Nonlinear metrics

#### 2.2.1 SD1, SD2, and SD1/SD2 ratio

The Poincare plot standard deviation (SD1 and SD2) is calculated by first fitting an ellipse to the plotted RR intervals. SD1 represents the standard deviation of the distance of each point to perpendicular line of identity, whereas SD2 represents the standard deviation from each point to the fitted curve *y* = *x*. Mathematically, these are represented as

$$\left[{\mathrm{RR}}_{x}\right]=\left[{\mathrm{RR}}_{1},{\mathrm{RR}}_{2},\ldots ,{\mathrm{RR}}_{N-1}\right]$$

$$\left[{\mathrm{RR}}_{y}\right]=\left[{\mathrm{RR}}_{2},{\mathrm{RR}}_{3},\ldots ,{\mathrm{RR}}_{N}\right]$$

$$\mathrm{SD}1=\mathrm{SD}\left(\frac{{\mathrm{RR}}_{x}-{\mathrm{RR}}_{y}}{\sqrt{2}}\right)$$

$$\mathrm{SD}2=\mathrm{SD}\left(\frac{{\mathrm{RR}}_{x}+{\mathrm{RR}}_{y}}{\sqrt{2}}\right)$$

$$\mathrm{SD}1/\mathrm{SD}2=\frac{\mathrm{SD}1}{\mathrm{SD}2}$$

SD, sample standard deviation. ${\mathrm{RR}}_{x}\pm {\mathrm{RR}}_{y}$, element‐wise operation of the RR interval arrays. It is important to note that Poincaré plots are related to the linear indexes of HRV, such as SDNN and the standard deviation of successive differences (SDSD; Brennan et al., 2001). Nevertheless, these three metrics (SD1, SD2, and SD1/SD2) are still commonly used in HRV analysis for their computation simplicity and effectiveness (Sassi et al., 2015; Shaffer & Ginsberg, 2017). Similar to the time‐domain variables, data collection at less than 5 min in length shows some promise in drawing conclusions using SD2 and the SD1/SD2 ratio (Castaldo et al., 2019; Tulppo et al., 1996).

#### 2.2.2 Detrended fluctuation analysis

Detrended fluctuation analysis (DFA α1 and α2) is calculated by first dividing the data into segments of a given window length before subtracting the linear fit from each window. The fluctuation, F(n), calculates the root mean square of the detrended time series by

$$F\left(n\right)=\sqrt{\frac{1}{N}\sum_{k=1}^{N}\;{\left\langle y\left(k\right)-y_{n}\left(k\right)\right\rangle }^{2}}$$

where *N* is the length of the time series, $y_{n}\left(k\right)$ is the local trend within each window, and $y\left(k\right)$ is the value of the integrated time series (Peng et al., 1995). The scaling component, DFA α1, is the ratio of *F*(*n*) over window sizes (*n* = 4–16) on a log–log plot. Shaffer et al. (2016) found that DFA α1 from 120 s of recording and DFA α2 from 180 s of recording could accurately represent 5‐min recordings.

#### 2.2.3 Statistical and data analysis

We first compared each activity at the 2‐min resolution. That is, we compared the first 2‐min segment of viewing to first 2‐min segment of tasks, in which that task segment was compared to the second 2‐min segment of viewing, etc., for a total of 17 comparisons. In addition, the viewing and the task segments were compared to their subsequent equivalent segments. That is, the first segment of viewing was compared to the second segment of viewing, etc.; the first segment of task was compared to the second segment of task, etc. We then investigated the data on a 1‐min resolution. That is, the first min of the first segment of viewing was compared to the second min of the first segment of viewing, and the second minute of the first segment of viewing was compared to the first min of the first segment of task, and so on, for 35 pairs of comparisons total. Paired sample t‐test analysis was performed to all pairings using SPSS, version 28.0 (IBM SPSS Statistics for Macintosh). RR interval extraction and the six HRV metrics variable computation were done using MATLAB, version 9.11.0 (MATLAB 2021b, The MathWorks, Inc). Figures were generated using R, version 4.1.3 (R Core Team, 2022) and ggplot2, version 3.4.1 (Wickham, 2016).

## 3 RESULTS

Participants assigned to the pure image regime presented no significant differences when compared to those assigned to the mixed image regime through any of the six metrics during viewing. SDNN, for example, in the pure regime participants ranged from 43.4 (SD = 17.1) to 55.0 (SD = 19.8), whereas mixed regime participants ranged from 56.4 (SD = 24.5) to 64.1 (SD = 29.1; Tables S1–S8; Figures S1–S6). Multiple hypothesis testing corrected the significance level to *p* < 0.0056 (Bonferroni correction). The following results are presented for all participants from both regimes via paired results of our data, specifically looking at changes in HRV within each participant over the course of the study.

**FIGURE 2:**
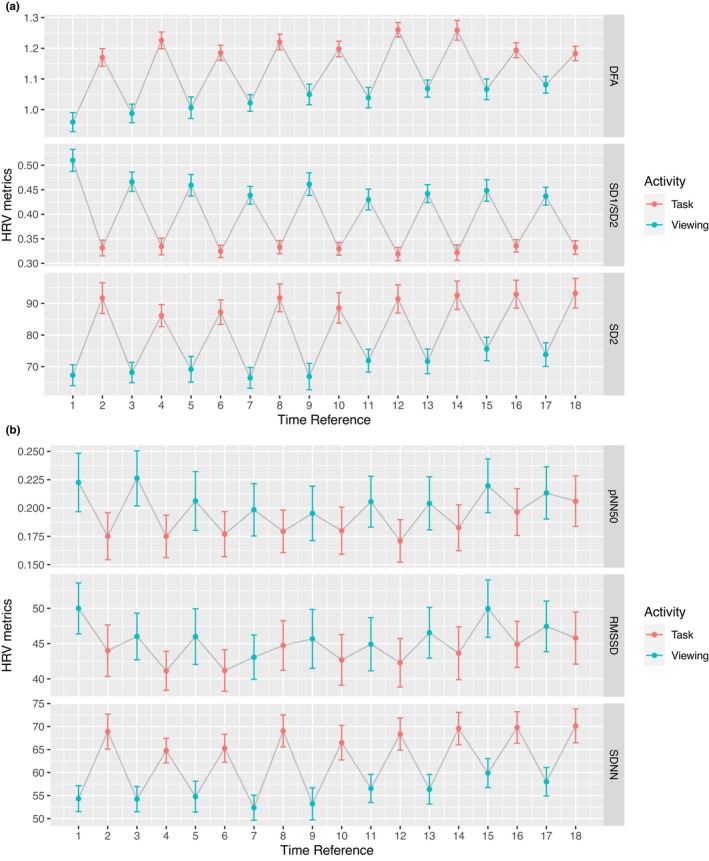
HRV metrics by segments for the 2‐min resolution. Odd numbered Time References represent Viewing; even numbered Time References represent Task. The gray line connects the activities chronologically. (a) Time‐domain metrics. (b) Nonlinear metrics. DFA, detrended fluctuation analysis. SD1, standard deviation of the points perpendicular to the line of identity on a Poincaré plot. SD2, standard deviation of the points along the line of identity on a Poincaré plot. pNN50, percentage of subsequent RR intervals differing more than 50 ms. RMSSD, the root mean square of successive RR interval differences. SDNN, the standard deviation of normal‐to‐normal beats. Viewing: participants view images from a balanced pool. Tasks: participants perform an arithmetic test and recall the previously seen images.

For comparisons within a subject between each activity to its subsequent activity on the 2‐min resolution, SDNN, SD1 to SD2 ratio, SD2, and DFA showed significances in all 17 pairs, while pNN50 showed significances in 1 pair and RMSSD showed significances in 0 pairs (Tables 1 and 2). SDNN ranged from 52.4 (SD = 22.5) to 59.9 (SD = 25.4) during viewing, and from 64.8 (SD = 20.5) to 70.1 (SD = 28.3) during tasks. SD1 to SD2 ratio ranged from 0.43 (SD = 0.17) to 0.51 (SD = 0.18) during viewing, and from 0.32 (SD = 0.11) to 0.34 (SD = 0.10) during tasks. DFA ranged from 0.96 (SD = 0.25) to 1.08 (SD = 0.22) during viewing and from 1.17 (SD = 0.21) to 1.26 (SD = 0.25) during tasks. pNN50 ranged from 0.20 (SD = 0.19) to 0.23 (SD = 0.20) during viewing, and from 0.17 (SD = 0.15) to 0.21 (SD = 0.17) during tasks. SD2 ranged from 66.42 (SD = 27.19) to 75.57 (SD = 30.01) during viewing, and from 86.16 (SD = 26.92) to 93.25 (SD = 35.86) during tasks. RMSSD ranged from 43.07 (SD = 25.89) to 49.99 (SD = 28.74) during viewing, and from 41.14 (SD = 21.20) to 45.78 (SD = 28.32) during tasks. The progression of an emergent alternating pattern in each HRV metrics over time for the 2‐min resolution is shown in Figure 2.

**TABLE 1:** Time‐domain metrics 2‐min comparison. *p*‐values are reported in comparison with previous activity. All numbers except *p*‐values are presented as mean (SD).

	SDNN	*p*	RMSSD	*p*	pNN50	*p*
Viewing 1	54.3 (22.3)	—	50.0 (28.7)	—	0.22 (0.20)	—
Task 1	68.9 (28.2)	<0.0029	44.0 (27.0)	0.212	0.18 (0.15)	0.016
Viewing 2	54.2 (22.5)	<0.0029	46.0 (27.2)	0.951	0.23 (0.20)	0.008
Task 2	64.8 (20.5)	<0.0029	41.1 (21.2)	0.043	0.17 (0.14)	<0.0029
Viewing 3	54.8 (25.1)	<0.0029	46.0 (29.5)	0.290	0.21 (0.19)	0.035
Task 3	65.3 (23.4)	<0.0029	41.2 (22.8)	0.172	0.18 (0.15)	0.056
Viewing 4	52.4 (22.5)	<0.0029	43.1 (25.9)	0.717	0.20 (0.19)	0.129
Task 4	69.1 (27.3)	<0.0029	44.7 (27.7)	0.618	0.18 (0.15)	0.275
Viewing 5	53.2 (27.0)	<0.0029	45.7 (32.3)	0.970	0.20 (0.19)	0.292
Task 5	66.5 (28.8)	<0.0029	42.7 (27.5)	0.298	0.18 (0.16)	0.152
Viewing 6	56.5 (25.0)	<0.0029	44.9 (30.9)	0.536	0.21 (0.18)	0.012
Task 6	68.4 (27.4)	<0.0029	42.3 (27.0)	0.782	0.17 (0.15)	0.056
Viewing 7	56.4 (26.5)	<0.0029	46.5 (29.6)	0.634	0.20 (0.19)	0.033
Task 7	69.6 (26.8)	<0.0029	43.6 (28.5)	0.673	0.18 (0.15)	0.120
Viewing 8	59.9 (25.4)	<0.0029	50.0 (32.8)	0.399	0.22 (0.19)	0.141
Task 8	69.8 (26.7)	<0.0029	44.9 (25.5)	0.121	0.20 (0.16)	0.029
Viewing 9	58.0 (25.5)	<0.0029	47.4 (29.5)	0.318	0.21 (0.19)	0.010
Task 9	70.1 (28.3)	<0.0029	45.8 (28.3)	0.208	0.21 (0.17)	0.010

**TABLE 2:** Nonlinear metrics 2‐min comparison. *p*‐values are reported in comparison with previous activity. All numbers except *p*‐values are presented as mean (SD).

	SD1/SD2	*p*	SD2	*p*	DFA	*p*
Viewing 1	0.51 (0.18)	—	67.3 (26.3)	—	0.96 (0.25)	—
Task 1	0.33 (0.12)	<0.0029	91.7 (36.2)	<0.0029	1.17 (0.21)	<0.0029
Viewing 2	0.47 (0.16)	<0.0029	68.2 (26.7)	<0.0029	0.99 (0.25)	<0.0029
Task 2	0.33 (0.13)	<0.0029	86.2 (26.9)	<0.0029	1.23 (0.21)	<0.0029
Viewing 3	0.46 (0.16)	<0.0029	69.1 (30.5)	<0.0029	1.01 (0.26)	<0.0029
Task 3	0.32 (0.10)	<0.0029	87.2 (30.1)	<0.0029	1.19 (0.19)	<0.0029
Viewing 4	0.44 (0.15)	<0.0029	66.4 (27.2)	<0.0029	1.02 (0.22)	<0.0029
Task 4	0.33 (0.11)	<0.0029	91.8 (34.6)	<0.0029	1.22 (0.20)	<0.0029
Viewing 5	0.46 (0.18)	<0.0029	66.9 (32.4)	<0.0029	1.05 (0.26)	<0.0029
Task 5	0.33 (0.10)	<0.0029	88.6 (37.0)	<0.0029	1.20 (0.20)	<0.0029
Viewing 6	0.43 (0.17)	<0.0029	71.9 (29.6)	<0.0029	1.04 (0.27)	<0.0029
Task 6	0.32 (0.11)	<0.0029	91.4 (35.3)	<0.0029	1.26 (0.19)	<0.0029
Viewing 7	0.44 (0.15)	<0.0029	71.7 (32.3)	<0.0029	1.07 (0.23)	<0.0029
Task 7	0.32 (0.12)	<0.0029	92.6 (34.1)	<0.0029	1.26 (0.25)	<0.0029
Viewing 8	0.45 (0.18)	<0.0029	75.6 (30.0)	<0.0029	1.07 (0.27)	<0.0029
Task 8	0.34 (0.10)	<0.0029	92.9 (34.6)	<0.0029	1.19 (0.19)	<0.0029
Viewing 9	0.44 (0.15)	<0.0029	73.8 (30.8)	<0.0029	1.08 (0.22)	<0.0029
Task 9	0.33 (0.11)	<0.0029	93.3 (35.9)	<0.0029	1.18 (0.18)	<0.0029

Comparing segments within the same activity on the 2‐min resolution (i.e., viewing vs. viewing or tasks vs. tasks), SD1 to SD2 ratio showed significances in one pair. The remaining five metrics showed significances in 0 pairs (Tables 3 and 4). Multiple hypothesis testing corrected the significance level in this resolution to *p* < 0.0029 (Bonferroni correction).

**TABLE 3:** Time‐domain metrics 2‐min comparison within tasks and within viewing. *p*‐values are reported in comparison with previous activity. All numbers except *p*‐values are presented as mean (SD).

	SDNN	*p*	RMSSD	*p*	pNN50	*p*
*Viewing*						
Viewing 1	54.3 (22.3)	—	50.0 (28.7)	—	0.22 (0.20)	—
Viewing 2	54.2 (22.5)	0.948	46.0 (27.2)	0.049	0.23 (0.20)	0.906
Viewing 3	54.8 (25.1)	0.906	46.0 (29.5)	0.959	0.21 (0.19)	0.048
Viewing 4	52.4 (22.5)	0.386	43.1 (25.9)	0.215	0.20 (0.19)	0.880
Viewing 5	53.2 (27.0)	0.626	45.7 (32.3)	0.264	0.20 (0.19)	0.719
Viewing 6	56.5 (25.0)	0.141	44.9 (30.9)	0.883	0.21 (0.18)	0.101
Viewing 7	56.4 (26.5)	0.977	46.5 (29.6)	0.445	0.20 (0.19)	0.894
Viewing 8	59.9 (25.4)	0.282	50.0 (32.8)	0.366	0.22 (0.19)	0.261
Viewing 9	58.0 (25.5)	0.635	47.4 (29.5)	0.663	0.21 (0.19)	0.961
*Task*						
Task 1	68.9 (28.2)	—	44.0 (27.0)	—	0.18 (0.15)	—
Task 2	64.8 (20.5)	0.282	41.1 (21.2)	0.436	0.17 (0.14)	0.956
Task 3	65.3 (23.4)	0.458	41.2 (22.8)	0.651	0.18 (0.15)	0.429
Task 4	69.1 (27.3)	0.191	44.7 (27.7)	0.117	0.18 (0.15)	0.925
Task 5	66.5 (28.8)	0.235	42.7 (27.5)	0.393	0.18 (0.16)	0.888
Task 6	68.4 (27.4)	0.088	42.3 (27.0)	0.167	0.17 (0.15)	0.818
Task 7	69.6 (26.8)	0.318	43.6 (28.5)	0.305	0.18 (0.15)	0.110
Task 8	69.8 (26.7)	0.204	44.9 (25.5)	0.183	0.20 (0.16)	0.966
Task 9	70.1 (28.3)	0.811	45.8 (28.3)	0.921	0.21 (0.17)	0.692

**TABLE 4:** Nonlinear metrics 2‐min comparison within tasks and within viewing. *p*‐values are reported in comparison with previous activity. All numbers except *p*‐values are presented as mean (SD).

	SD1/SD2	*p*	SD2	*p*	DFA	*p*
*Viewing*						
Viewing 1	0.51 (0.18)	—	67.3 (26.3)	—	0.96 (0.25)	—
Viewing 2	0.47 (0.16)	<0.0029	68.2 (26.7)	0.459	0.99 (0.25)	0.151
Viewing 3	0.46 (0.16)	0.838	69.1 (30.5)	0.842	1.01 (0.26)	0.581
Viewing 4	0.44 (0.15)	0.093	66.4 (27.2)	0.562	1.02 (0.22)	0.495
Viewing 5	0.46 (0.18)	0.155	66.9 (32.4)	0.849	1.05 (0.26)	0.160
Viewing 6	0.43 (0.17)	0.223	71.9 (29.6)	0.059	1.04 (0.27)	0.249
Viewing 7	0.44 (0.15)	0.518	71.7 (32.3)	0.989	1.07 (0.23)	0.416
Viewing 8	0.45 (0.18)	0.713	75.6 (30.0)	0.407	1.07 (0.27)	0.631
Viewing 9	0.44 (0.15)	0.759	73.8 (30.8)	0.728	1.08 (0.22)	0.532
*Task*						
Task 1	0.33 (0.12)	—	91.7 (36.2)	—	1.17 (0.21)	—
Task 2	0.33 (0.13)	0.609	86.2 (26.9)	0.295	1.23 (0.21)	0.171
Task 3	0.32 (0.10)	0.572	87.2 (30.1)	0.424	1.19 (0.19)	0.057
Task 4	0.33 (0.11)	0.569	91.8 (34.6)	0.313	1.22 (0.20)	0.062
Task 5	0.33 (0.10)	0.978	88.6 (37.0)	0.276	1.20 (0.20)	0.133
Task 6	0.32 (0.11)	0.747	91.4 (35.3)	0.108	1.26 (0.19)	0.036
Task 7	0.32 (0.12)	0.984	92.6 (34.1)	0.413	1.26 (0.25)	0.799
Task 8	0.34 (0.10)	0.735	92.9 (34.6)	0.327	1.19 (0.19)	0.279
Task 9	0.33 (0.11)	0.580	93.3 (35.9)	0.807	1.18 (0.18)	0.812

**FIGURE 3:**
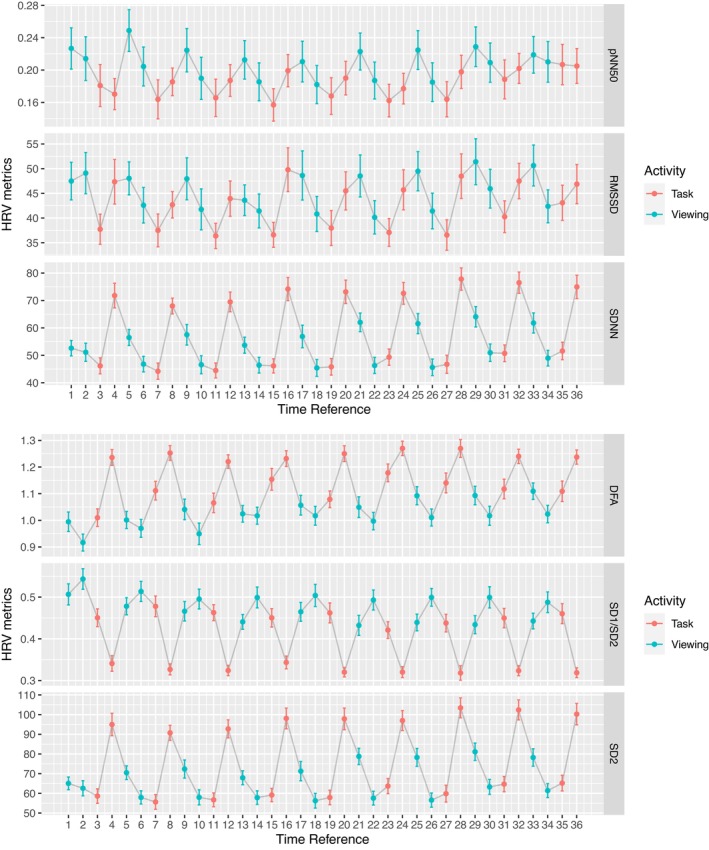
HRV metrics by segments for the 1‐min resolution. Time Reference 1: first 60s of viewing. Time reference 2: second 60s of viewing. Time Reference 3: arithmetic tests. Time Reference 4: recalling the images presented during the segment. This pattern continued for the remaining Time References. (a) time‐domain metrics. (b) nonlinear metrics.

Comparing within a subject between each minute of the activity to its subsequent minute presented significant differences, though unevenly, throughout the six metrics. Out of the 35 total pairs of comparison, SDNN presented 23 pairs of significances, RMSSD presented four pairs, pNN50 presented seven pairs, SD1 to SD2 ratio presented 19 pairs, SD2 presented 25 pairs, and DFA presented 15 pairs (Tables 5 and 6). We break down this result by comparing segments within the same activity on the 1‐min resolution. Comparing the tasks on a 1‐min resolution (i.e., the second minute of task to first minute of task), SDNN, pNN50, SD1/SD2, and SD2 presented nine significant HRV differences out of nine total comparisons. DFA showed significance for five out of nine for such comparisons. RMSSD presented three out of nine significant comparisons (Tables 7 and 8). Comparing the viewing on a 1‐min resolution, (i.e., the second minute of viewing to first minute of viewing), SD2 and SDNN presented eight significant HRV differences out of nine total comparisons. pNN50 presented five out of nine for such comparisons. RMSSD presented one out of nine for such comparisons. SD1 to SD2 ratio and DFA presented 0 out of 9 for such comparisons (Tables 9 and 10). Multiple hypothesis testing corrected the significance level at this resolution to *p* < 0.0014 (Bonferroni correction). The progression of the alternating pattern in each HRV metrics over time on the 1‐min resolution is shown in Figure 3.

**TABLE 5:** Time‐domain metrics 1‐min comparison within tasks. All numbers except *p*‐values are presented as mean (SD).

	SDNN	*p*	RMSSD	*p*	pNN50	*p*
Task 1 min 1	46.2 (21.7)	—	37.7 (22.7)	—	0.18 (0.19)	—
Task 1 min 2	71.8 (33.4)	<0.0014	47.3 (33.5)	0.013	0.17 (0.14)	<0.0014
Task 2 min 1	44.2 (22.8)	—	37.5 (25.5)	—	0.16 (0.19)	—
Task 2 min 2	68.0 (22.1)	<0.0014	42.7 (20.5)	0.062	0.19 (0.13)	<0.0014
Task 3 min 1	44.5 (21.0)	—	36.4 (19.8)	—	0.17 (0.18)	—
Task 3 min 2	69.5 (27.9)	<0.0014	43.9 (27.6)	0.012	0.19 (0.15)	<0.0014
Task 4 min 1	46.2 (20.4)	—	36.6 (19.9)	—	0.16 (0.16)	—
Task 4 min 2	74.2 (33.1)	<0.0014	49.8 (34.9)	<0.0014	0.20 (0.16)	<0.0014
Task 5 min 1	45.8 (23.2)	—	38.0 (27.2)	—	0.17 (0.17)	—
Task 5 min 2	73.1 (32.8)	<0.0014	45.5 (29.7)	0.011	0.19 (0.16)	<0.0014
Task 6 min 1	49.3 (23.3)	—	37.1 (22.1)	—	0.16 (0.16)	—
Task 6 min 2	72.6 (31.4)	<0.0014	45.7 (32.0)	<0.0014	0.18 (0.15)	<0.0014
Task 7 min 1	46.7 (25.0)	—	36.6 (23.4)	—	0.16 (0.17)	—
Task 7 min 2	77.8 (30.9)	<0.0014	48.5 (34.4)	<0.0014	0.20 (0.16)	<0.0014
Task 8 min 1	50.7 (23.7)	—	40.3 (25.1)	—	0.19 (0.19)	—
Task 8 min 2	76.5 (30.3)	<0.0014	47.5 (27.9)	0.005	0.20 (0.14)	<0.0014
Task 9 min 1	51.6 (24.5)	—	43.1 (27.4)	—	0.21 (0.19)	—
Task 9 min 2	75.0 (32.6)	<0.0014	46.9 (30.6)	0.109	0.20 (0.17)	<0.0014

**TABLE 6:** Nonlinear metrics 1‐min comparison within tasks. All numbers except *p*‐values are presented as mean (SD).

	SD1/SD2	*p*	SD2	*p*	DFA	*p*
Task 1 min 1	0.45 (0.16)	—	58.6 (27.3)	—	1.01 (0.24)	—
Task 1 min 2	0.34 (0.14)	<0.0014	95.0 (42.2)	<0.0014	1.25 (0.22)	<0.0014
Task 2 min 1	0.48 (0.19)	—	55.6 (28.6)	—	1.11 (0.27)	—
Task 2 min 2	0.33 (0.10)	<0.0014	90.7 (29.3)	<0.0014	1.25 (0.20)	<0.0014
Task 3 min 1	0.46 (0.15)	—	56.7 (27.1)	—	1.07 (0.28)	—
Task 3 min 2	0.32 (0.10)	<0.0014	92.7 (35.5)	<0.0014	1.22 (0.20)	<0.0014
Task 4 min 1	0.45 (0.17)	—	59.1 (26.6)	—	1.15 (0.32)	—
Task 4 min 2	0.34 (0.12)	<0.0014	98.0 (41.5)	<0.0014	1.22 (0.22)	0.0846
Task 5 min 1	0.46 (0.18)	—	57.9 (28.3)	—	1.08 (0.24)	—
Task 5 min 2	0.32 (0.09)	<0.0014	97.8 (42.2)	<0.0014	1.24 (0.22)	<0.0014
Task 6 min 1	0.42 (0.16)	—	63.7 (30.4)	—	1.18 (0.26)	—
Task 6 min 2	0.32 (0.10)	<0.0014	97.0 (39.8)	<0.0014	1.27 (0.20)	0.0036
Task 7 min 1	0.44 (0.16)	—	59.8 (32.4)	—	1.14 (0.28)	—
Task 7 min 2	0.32 (0.13)	<0.0014	103.4 (38.6)	<0.0014	1.26 (0.23)	<0.0014
Task 8 min 1	0.45 (0.18)	—	64.7 (30.3)	—	1.12 (0.29)	—
Task 8 min 2	0.32 (0.09)	<0.0014	102.4 (39.7)	<0.0014	1.22 (0.19)	0.0018
Task 9 min 1	0.46 (0.18)	—	65.2 (30.4)	—	1.11 (0.29)	—
Task 9 min 2	0.32 (0.09)	<0.0014	100.2 (41.8)	<0.0014	1.21 (0.20)	0.0067

**TABLE 7:** Time‐domain metrics 1‐min comparison within viewing. All numbers except *p*‐values are presented as mean (SD).

	SDNN	*p*	RMSSD	*p*	pNN50	*p*
Viewing 1 min 1	52.6 (22.2)	—	47.5 (30.3)	—	0.23 (0.20)	—
Viewing 1 min 2	51.1 (26.0)	0.572	49.1 (32.9)	0.640	0.21 (0.21)	0.457
Viewing 2 min 1	56.5 (24.0)	—	48.0 (27.5)	—	0.25 (0.21)	—
Viewing 2 min 2	46.8 (23.4)	<0.0014	42.6 (29.7)	0.033	0.20 (0.20)	<0.0014
Viewing 3 min 1	57.5 (28.0)	—	47.9 (31.9)	—	0.22 (0.20)	—
Viewing 3 min 2	46.6 (24.5)	<0.0014	41.7 (31.0)	0.142	0.19 (0.19)	<0.0014
Viewing 4 min 1	53.7 (24.0)	—	43.6 (25.7)	—	0.21 (0.20)	—
Viewing 4 min 2	46.4 (23.5)	<0.0014	41.4 (28.3)	0.367	0.19 (0.19)	0.003
Viewing 5 min 1	56.9 (31.9)	—	48.6 (38.5)	—	0.21 (0.19)	—
Viewing 5 min 2	45.4 (23.8)	<0.0014	40.8 (27.6)	0.037	0.18 (0.18)	<0.0014
Viewing 6 min 1	62.0 (27.8)	—	48.5 (34.8)	—	0.22 (0.19)	—
Viewing 6 min 2	46.3 (24.1)	<0.0014	40.1 (27.6)	<0.0014	0.19 (0.19)	<0.0014
Viewing 7 min 1	61.6 (30.2)	—	49.5 (32.7)	—	0.22 (0.20)	—
Viewing 7 min 2	45.6 (25.0)	<0.0014	41.4 (29.7)	0.009	0.19 (0.20)	<0.0014
Viewing 8 min 1	64.1 (29.9)	—	51.4 (37.5)	—	0.23 (0.20)	—
Viewing 8 min 2	50.9 (25.6)	<0.0014	45.9 (31.6)	0.147	0.21 (0.20)	0.090
Viewing 9 min 1	61.8 (30.1)	—	50.6 (34.2)	—	0.22 (0.19)	—
Viewing 9 min 2	49.0 (23.6)	<0.0014	42.4 (27.4)	0.010	0.21 (0.21)	0.456

**TABLE 8:** Nonlinear metrics 1‐min comparison within viewing. All numbers except *p*‐values are presented as mean (SD).

	SD1/SD2	*p*	SD2	*p*	DFA	*p*
Viewing 1 min 1	0.51 (0.20)	—	65.1 (25.6)	—	0.99 (0.29)	—
Viewing 1 min 2	0.54 (0.19)	0.117	62.6 (30.4)	0.355	0.91 (0.27)	0.008
Viewing 2 min 1	0.48 (0.17)	—	70.5 (28.8)	—	1.00 (0.26)	—
Viewing 2 min 2	0.51 (0.20)	0.051	57.9 (27.6)	<0.0014	0.97 (0.26)	0.285
Viewing 3 min 1	0.47 (0.17)	—	72.4 (34.3)	—	1.04 (0.29)	—
Viewing 3 min 2	0.50 (0.18)	0.235	58.0 (28.6)	<0.0014	0.97 (0.3)	0.053
Viewing 4 min 1	0.44 (0.15)	—	67.9 (29.3)	—	1.02 (0.26)	—
Viewing 4 min 2	0.50 (0.21)	0.007	57.8 (28.4)	<0.0014	1.01 (0.27)	0.639
Viewing 5 min 1	0.46 (0.18)	—	71.3 (37.8)	—	1.06 (0.29)	—
Viewing 5 min 2	0.50 (0.21)	0.067	56.3 (28.9)	<0.0014	1.02 (0.28)	0.337
Viewing 6 min 1	0.43 (0.20)	—	78.8 (33.8)	—	1.05 (0.32)	—
Viewing 6 min 2	0.49 (0.20)	0.003	57.6 (28.6)	<0.0014	1.00 (0.27)	0.174
Viewing 7 min 1	0.44 (0.16)	—	78.3 (37.5)	—	1.09 (0.28)	—
Viewing 7 min 2	0.50 (0.18)	0.013	56.6 (29.7)	<0.0014	1.02 (0.28)	0.058
Viewing 8 min 1	0.43 (0.17)	—	81.1 (35.4)	—	1.09 (0.28)	—
Viewing 8 min 2	0.50 (0.21)	0.002	63.3 (30.5)	<0.0014	1.03 (0.28)	0.048
Viewing 9 min 1	0.44 (0.15)	—	78.2 (36.3)	—	1.11 (0.25)	—
Viewing 9 min 2	0.49 (0.20)	0.079	61.4 (29.2)	<0.0014	1.04 (0.28)	0.035

**FIGURE 4:**
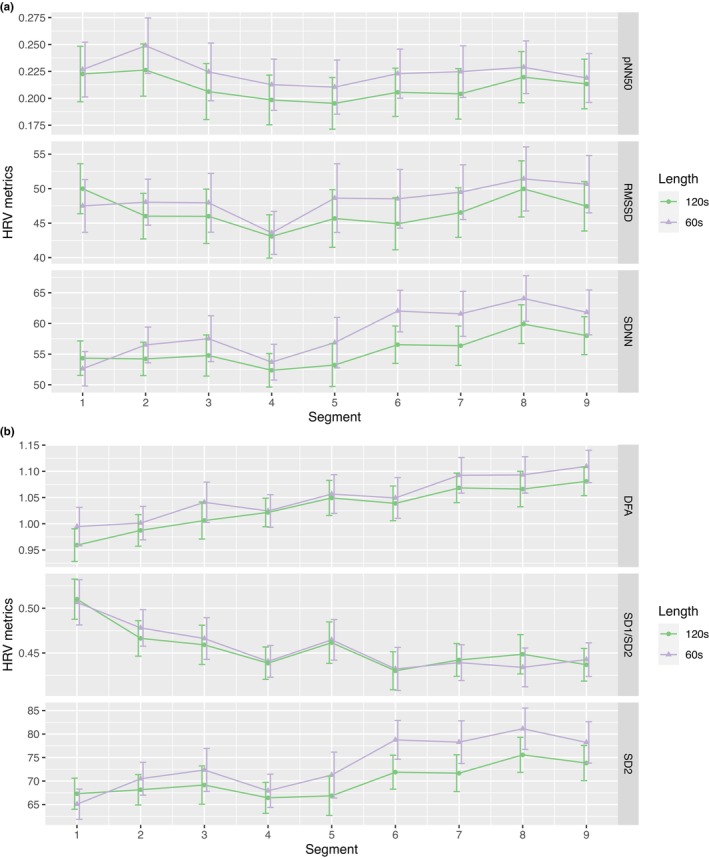
HRV metrics comparison during viewing for input sequence length of 60 and 120 s. 60s: the first 60s of viewing were used to compute each metric. 120 s: the first 120 s of viewing were used to compute each metric. (a) time‐domain metrics. (b) nonlinear metrics.

The analysis of various recording lengths (60s vs. 120s) revealed that pNN50 was the most sensitive measure in terms of efficacy as a function of data length, demonstrating statistical significance in six out of nine segments. SDNN and SD2 were also sensitive, albeit less so, showing statistical significance in three out of nine segments. RMSSD was significant in only one out of nine segments. In contrast, DFA and SD1/SD2 did not show statistical significance in any of the nine segments analyzed (Tables 9 and 10; Figure 4). Multiple hypothesis testing was performed to correct for the potential of false positives, and the significance level was set at *p* < 0.0029 (Bonferroni correction).

**TABLE 9:** Time‐domain metrics comparison during viewing for segment length of 60 and 120 s. *p*‐values are reported in comparison with previous activity. All numbers except *p*‐values are presented as mean (SD).

	ECG length (s)	SDNN	*p*	RMSSD	*p*	pNN50	*p*
Viewing 1	120	54.3 (22.3)	0.213	50.0 (4.0)	0.265	0.22 (0.20)	0.404
	60	52.6 (22.2)		47.0 (4.0)		0.23 (0.20)	
Viewing 2	120	54.2 (22.4)	0.031	46.0 (3.0)	0.123	0.23 (0.20)	<0.0029
	60	56.5 (24.0)		48.0 (3.0)		0.25 (0.21)	
Viewing 3	120	54.8 (25.1)	0.100	46.0 (4.0)	0.421	0.21 (0.19)	<0.0029
	60	57.5 (28.0)		48.0 (4.0)		0.22 (0.20)	
Viewing 4	120	52.4 (22.5)	0.176	43.0 (3.0)	0.707	0.20 (0.19)	<0.0029
	60	53.7 (24.0)		44.0 (3.0)		0.21 (0.20)	
Viewing 5	120	53.2 (27.0)	0.004	46.0 (4.0)	0.074	0.20 (0.19)	<0.0029
	60	56.9 (31.9)		49.0 (5.0)		0.21 (0.19)	
Viewing 6	120	56.5 (25.0)	<0.0029	45.0 (4.0)	<0.0029	0.21 (0.18)	<0.0029
	60	62.0 (27.8)		49.0 (4.0)		0.22 (0.19)	
Viewing 7	120	56.4 (26.4)	<0.0029	47.0 (4.0)	0.038	0.20 (0.19)	<0.0029
	60	61.6 (30.2)		49.0 (4.0)		0.22 (0.20)	
Viewing 8	120	59.9 (25.4)	0.009	50.0 (4.0)	0.493	0.22 (0.19)	0.115
	60	64.1 (29.9)		51.0 (5.0)		0.23 (0.20)	
Viewing 9	120	58.0 (25.5)	<0.0029	47.0 (4.0)	0.007	0.21 (0.19)	0.346
	60	61.8 (30.1)		51.0 (4.0)		0.22 (0.19)	

**TABLE 10:** Nonlinear metrics comparison during viewing for segment length of 60s and 120 s. *p*‐values are reported in comparison with previous activity. All numbers except *p*‐values are presented as mean (SD).

	ECG length (s)	SD1/SD2	*p*	SD2	*p*	DFA	*p*
Viewing 1	120	0.51 (0.18)	0.827	67.3 (26.3)	0.133	0.96 (0.25)	0.053
	60	0.51 (0.20)		65.1 (25.6)		0.99 (0.29)	
Viewing 2	120	0.47 (0.16)	0.188	68.2 (26.7)	0.063	0.99 (0.25)	0.227
	60	0.48 (0.17)		70.5 (28.8)		1.00 (0.26)	
Viewing 3	120	0.46 (0.16)	0.622	69.2 (30.5)	0.084	1.01 (0.26)	0.155
	60	0.47 (0.17)		72.4 (34.3)		1.04 (0.29)	
Viewing 4	120	0.44 (0.15)	0.855	66.4 (27.2)	0.222	1.02 (0.22)	0.842
	60	0.44 (0.15)		67.9 (29.3)		1.02 (0.26)	
Viewing 5	120	0.46 (0.18)	0.762	66.9 (32.4)	0.004	1.05 (0.26)	0.595
	60	0.47 (0.18)		71.3 (37.8)		1.06 (0.29)	
Viewing 6	120	0.43 (0.17)	0.781	71.9 (29.6)	<0.0029	1.04 (0.27)	0.383
	60	0.43 (0.20)		78.8 (33.8)		1.05 (0.32)	
Viewing 7	120	0.44 (0.15)	0.761	71.7 (32.3)	<0.0029	1.07 (0.23)	0.110
	60	0.44 (0.16)		78.3 (37.5)		1.09 (0.28)	
Viewing 8	120	0.45 (0.18)	0.233	75.6 (30.0)	<0.0029	1.07 (0.27)	0.123
	60	0.43 (0.17)		81.1 (35.4)		1.09 (0.28)	
Viewing 9	120	0.44 (0.15)	0.475	73.8 (30.8)	0.005	1.08 (0.22)	0.029
	60	0.44 (0.15)		78.2 (36.3		1.11 (0.25)	

## 4 DISCUSSION

In this study, 70 participants completed a series of activities that included viewing images, arithmetic testing, and memory recall. We analyzed the robustness of our findings on both a 1‐ and 2‐min reoslution. Specifically, we explored our data within each time resolutions for participants across activities to determine whether HRV metrics maintined their integrity. Our analysis revealed that each of the six metrics we used (SDNN, RMSSD, pNN50, SD1 to SD2 ratio, SD2, and DFA) was effective in capturing some aspect of the study variables, such as distinguishing between passive and active periods and different activity types. None of the variables could distinguish between image regime. However, aside from the ratio of SD1 to SD2, none of the other metrics were able to capture activity types and passive/active periods simultaneously. The remaining five metrics appear to reflect the impact of external stimuli in our participants with varying degrees of sensitivity. We also looked at each metric's value at different time lengths to see whether considerable differences occurred between a 1‐min data series compared to a 2‐min data series. We found that pNN50 was the most susceptible to input length and that pNN50 was one of the least able to detect active versus passive periods. Despite this, many of the six metrics were effective in capturing various aspects of our study variables.

Past studies reported contradicting results on whether UST HRV measurements can be reliably used to measure valance. For instance, Choi et al. found that RR intervals generated from a single image displayed for 6 s can classify valence and changes thereof (Choi et al., 2017). However, Schippers et al. rejected Choi et al.'s claim by stating RMSSD measured with a 30s moving window cannot discern between positive and negative emotions with IAPS images (Schippers et al., 2018). In line with Schippers et al.'s findings, our results indicate that UST HRV measurements, including time‐domain and nonlinear metrics, cannot differentiate valence changes induced by images. Compared to both Choi et al and Schippers et al., we used a larger collection of IAPS images (*n* = 198) with longer analysis window (60 and 120 s), with each image collection replicated nine times. However, all segments yielded similar, non‐significant results in distinguishing valence, which supports the recommendation of using a standard recording time of 5 min in studies identifying HRV variables that can distinguish vagal tone (Laborde et al., 2017).

At the 2‐min resolution, all nonlinear metrics and SDNN showed statistically significant differences between the viewing and task conditions. As illustrated in Figure 2, we expected our HRV metrics to significantly alternate to reflect the changes in the viewing and task segments. This result is consistently displayed across four of our six HRV variables (SDNN, DFA, SD1/SD2, and SD2). It is possible that pNN50 and RMSSD are not sensitive enough to catch this feature. This suggests that the HRV dynamics were responsive to the intrinsic content viewing conditions, possibly due to the more passive engagement during viewing and the more cognitive demands required during the tasks, in addition to the stress of social evaluation. The stress‐induced sympathetic activities persisted to distract, as we still find significance in the later segments. This suggests that participants did not become habituated by the study, suggesting consistent stress recovery exhibited by healthy adults. One important finding on the 2‐min resolution analysis is that the nonlinear metrics out‐performed the time‐domain metrics. One possible explanation is that the nonlinear metrics such as DFA provide a nested approach to handling variation; this could provide a scale invariant result which reduces the importance of data length. To address concerns about potential temporal or repetitive confounders (i.e., whether differences observed were due to the repetition of tasks and increased study time), we tested and found none of the equivalent segments were statistically equivalent from each other, as shown in Tables 3 and 4. That is, the first segment of tasks has the same distribution of HRV metrics as that of the second segment, and the second segment of tasks has the same distribution as that of the third segments, and so on. This finding was also true for the viewing segments.

This study design goes beyond comparing passive and active segments, as it also allows for comparisons between activity types within each segment. This means that participants were compared in terms of passively reviewing images, actively recalling images, or actively computing arithmetic. While we expect no differences in HRV metrics between the first and second minutes of viewing due to the balanced nature of the images in terms of arousal, valence, and relatedness, we do expect shifts in HRV metrics between the first and second minutes of tasks, as participants may experience physiological symptoms of stress from social evaluation during the second minute (Schwabe et al., 2012).

After finding that most of the HRV metrics (aside from pNN50 and RMSSD) performed in response to the external conditions of this experiment as our intuition would expect at the 2‐min resolution, we checked the same data on a 1‐min resolution to see whether the results were upheld on a shorter 1‐min time scale. The study found that the six HRV metrics showed varying degrees of reliability in detecting activity types on the 1‐min resolution. SDNN and SD2 revealed significant HRV differences in 8 out of 9 viewing segments, indicating a difference during the first and second minutes of viewing, as illustrated in Figure 3 and Table 7 and 8. This is contrary to what we expect since images were intentionally balanced based in arousal, valence, and relatedness. Thus, SDNN and SD2 did not perform well in this study on the 1‐min scale for the viewing segment. For RMSSD, six out of nine task segments did not show significant HRV differences, suggesting no physiological differences during the first and second minutes of tasks, as illustrated in Table 5. This is also contrary to what we expect, because actively recalling images is likely to place participants into an elevated stress level by evaluating their performance, while the arithmetic tests were likely less stressful because the performance is not graded based on accuracy. Such change in the autonomic nervous system by evoking both the sympathetic and the parasympathetic effects should be reflected by RMSSD and other metrics (Shaffer et al., 2014). On the contrary, the SD1/SD2 ratio metric revealed participants' HRV in ways that are consistent with our expectation in every condition. SD1/SD2 did not present significant HRV differences in any viewing segments but did so in all task segments. It is worth noting that while SD1 (the equivalent of RMSSD) did not present many significant findings in either viewing or task segments, the divisor (SD2) presented overwhelming significances in both. The quotient of these two was sufficient to capture the nuance between viewing and task segments. It should be noted that DFA achieved a similar effective performance in detecting changes of external stimuli as SD1/SD2. Taken together, these results suggest that for studies involving changing activity types in ultra‐short term (UST) recording sessions, it may be beneficial to analyze multiple HRV metrics, particularly SD1/SD2 and DFA, to achieve a more holistic understanding of physiological behaviors.

Finally, to explore potential explanations for why there are differences observed in SDNN, pNN50, and SD2 the three variables that performed well during the 2‐min viewing segment but did not perform well during the 1‐min viewing segments, we compared each HRV metric with the first 60 s of viewing versus the first 120 s of viewing. Of the nine segments analyzed, SDNN, pNN50, and SD2 presented the highest numbers of significant differences in three, six, and three segments, respectively (Figure 4, Tables 9, and 10). These results are consistent in that there could be a link for HRV metrics that presented significances on the 1‐min viewing resolution and those presented significances in varying lengths. We recommend extracting HRV metrics at different length first, preferably from time periods characterized by consistent physiological states. This allows us to observe any significant changes associated with these different lengths before proceeding to other analyses that involve comparing two groups. It is helpful to undertake this step before drawing any HRV‐related conclusions at UST lengths. That is, HRV‐related conclusions should be done on the same time scale, as comparing segments in different length across different physiological states might not yield meaningful HRV results. Furthermore, contrary to some recent studies (Areas et al., 2018; Salahuddin et al., 2007; Shaffer et al., 2016), our results suggest that these three metrics may not be robust in evaluating UST HRVs in replacement of the standard short‐term HRV (i.e., recording length of 5 min), as they could not be replaced by their shorter recording length counterpart. While recent studies have suggested reliability of using 30s/60s/120 s to evaluate short‐term HRV (Chen et al., 2021; Melo et al., 2020; Munoz et al., 2015; Nakamura et al., 2017), our results suggest some HRV methods may not be robust within UST recordings. Future work may explore the effectiveness of the emerging heart rate fragmentation (Costa et al., 2017, 2022) in the context of UST series. Nonetheless, this study underscores the diverse and complex nature of UST recordings and provides caution for researchers when evaluating HRV at a shorter time scale.

## 5 LIMITATION AND FUTURE DIRECTIONS

One of the limitations of our study is that 49 out of 70 participants are female. Studies have shown that females may have greater parasympathetic autonomic function than males (Abhishekh et al., 2013; Koenig & Thayer, 2016). In addition, due to the design of this study and the potential fatigue in participant engagement, we could not validate whether the HRV metrics assessed have representative and normative values in 5‐min recordings. In addition, future studies could investigate the effect of talking during the task portion of the study. It could be that the act of talking had an impact and not the stress. It would be interesting to control for this factor in future research.

## 6 CONCLUSION

The evaluation of HRV from dynamic and complex ECG signals involves numerous metrics, of which UST HRV metrics only make up a small portion. Our research identified significant variations in SDNN, RMSSD, pNN50, SD2, SD1/SD2, and DFA at different time intervals, including in the 2‐min and 1‐min resolutions. Our findings suggest that SDNN, pNN50, and SD2 are sensitive to the length of the recording. We find that SD1/SD2 and DFA may potentially provide insights into the balance between stress‐induced sympathetic activity and parasympathetic‐mediated relaxation during UST HRV analysis. In contrary, RMSSD may not be able to distinguish such activities. While UST HRV continues to offer a noninvasive and efficient method for assessing cardiovascular health, its viability requires extensive empirical research at a larger scale.

## AUTHOR CONTRIBUTIONS

Carolyn Martsberger conceived and designed research. Vanessa Zarubin collected original ECG data. Zifan Gu performed experiments and analyzed data. Carolyn Martsberger and Zifan Gu interpreted results of experiments. Zifan Gu prepared figures. Carolyn Martsberger and Zifan Gu drafted the manuscript. Zifan Gu, Vanessa Zarubin, and Carolyn Martsberger edited, revised, and approved the final version of the manuscript.

## CONFLICT OF INTEREST STATEMENT

None of the authors declare any conflicts of interest, financial or otherwise.

## ETHICS STATEMENT

All participants provided written informed consent, and procedures were approved by the Wofford College Institutional Review Board.

## Supporting information


Figure S1.

Figure S2.

Figure S3.

Figure S4.

Figure S5.

Figure S6.

Tables S1‐Table S8.

## Data Availability

Data will be made available upon reasonable request.
