# Supplementary figures and images for: The effectiveness of time domain and nonlinear heart rate variability metrics in ultra‐short time series
Source: Physiol Rep. 2023 Nov 27;11(22):e15863. doi: 10.14814/phy2.15863 (PMC10681424; doi:10.14814/phy2.15863)

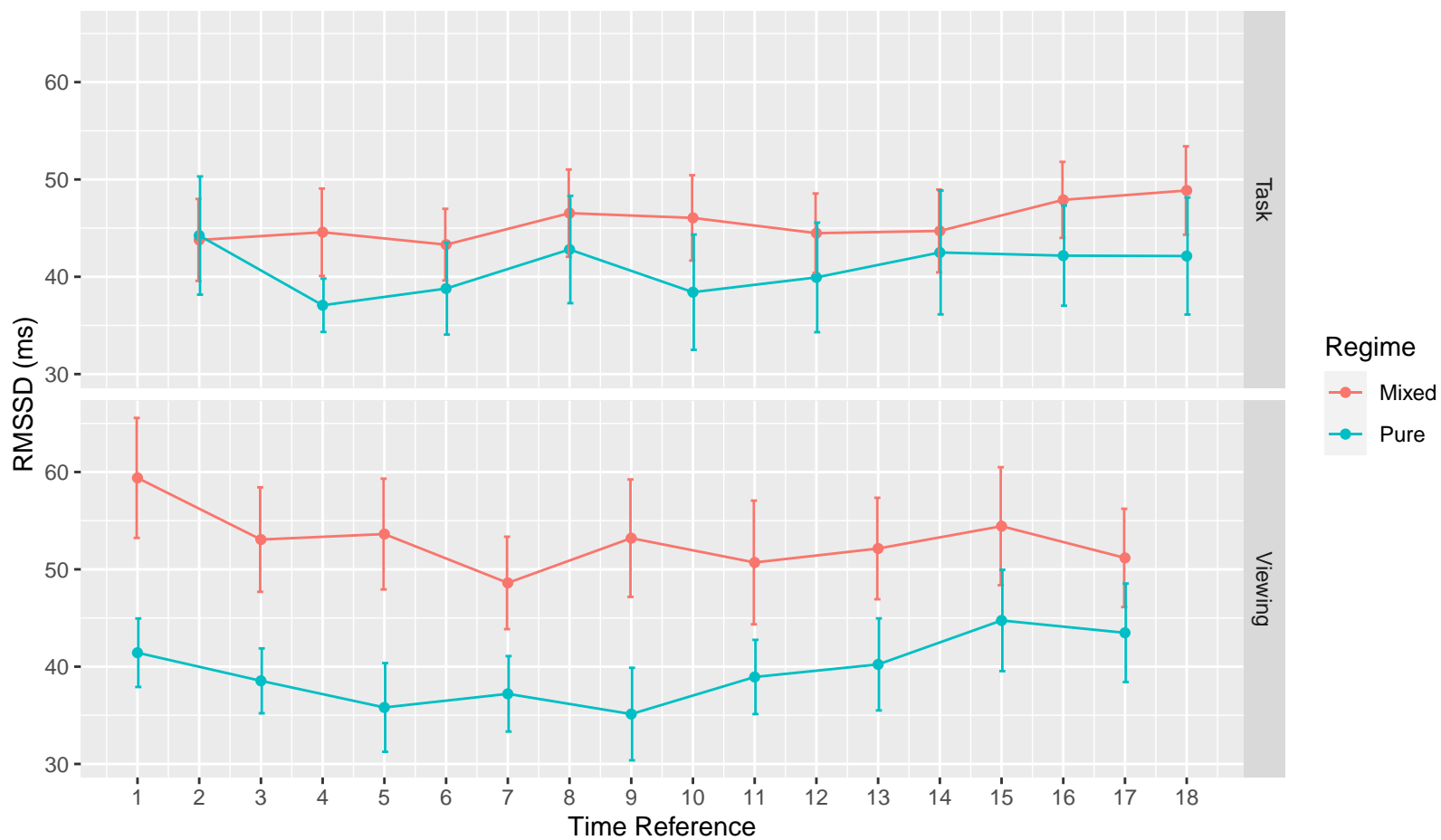

Supplement: Supplementary file 1 — Figure S1. [file PHY2-11-e15863-s002.pdf]

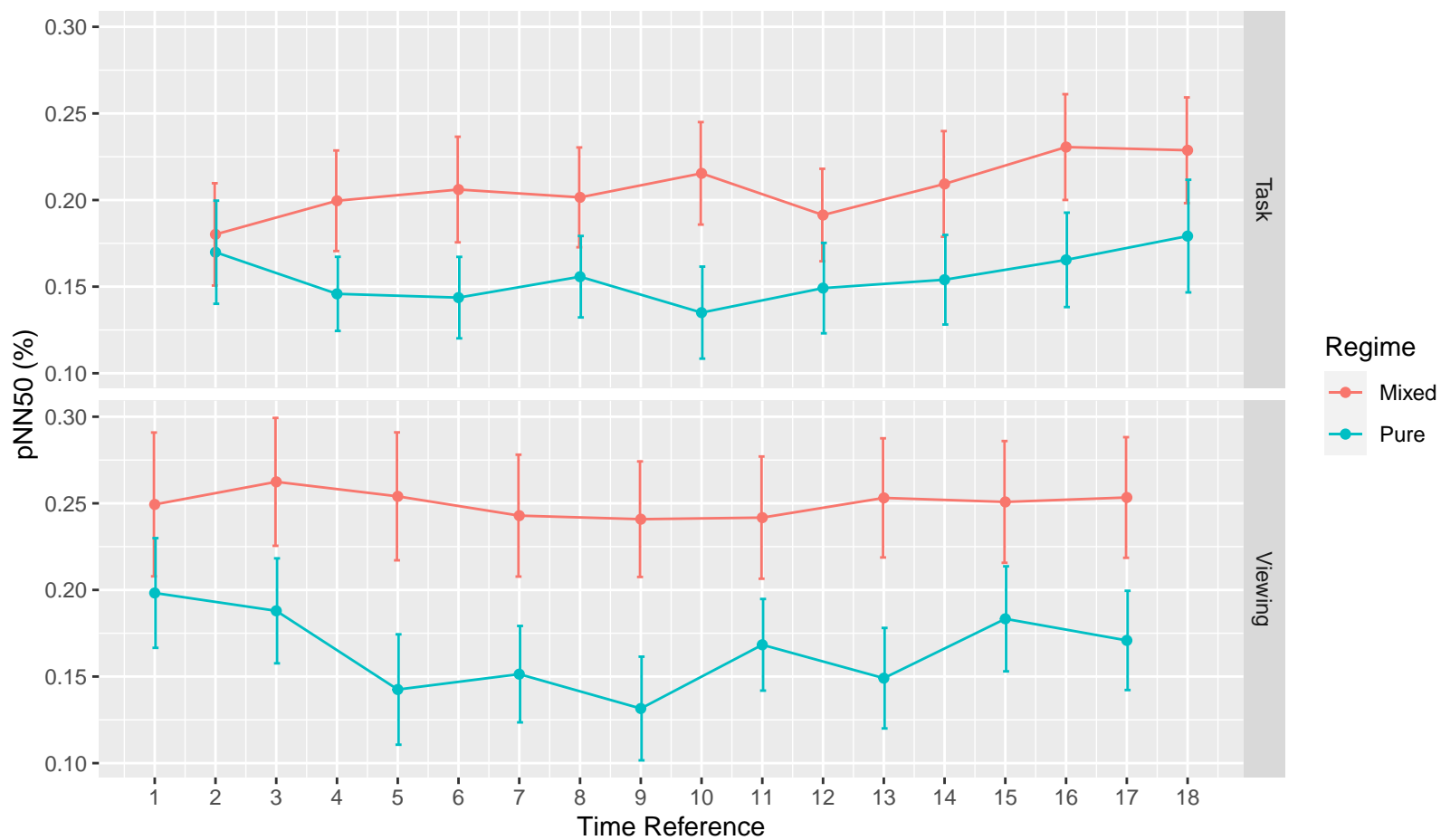

Supplement: Supplementary file 2 — Figure S2. [file PHY2-11-e15863-s007.pdf]

SD2

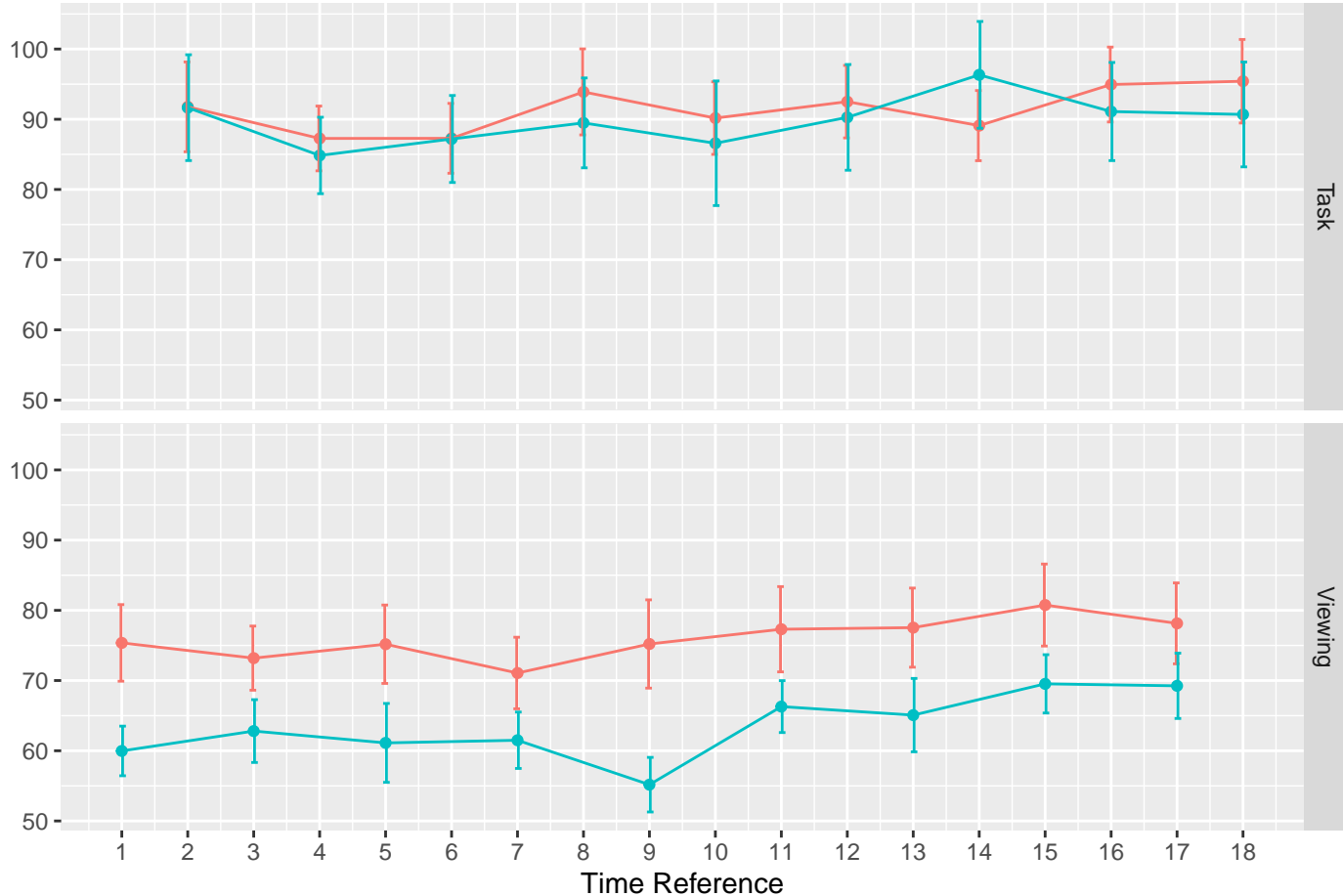

Supplement: Supplementary file 3 — Figure S3. [file PHY2-11-e15863-s006.pdf]

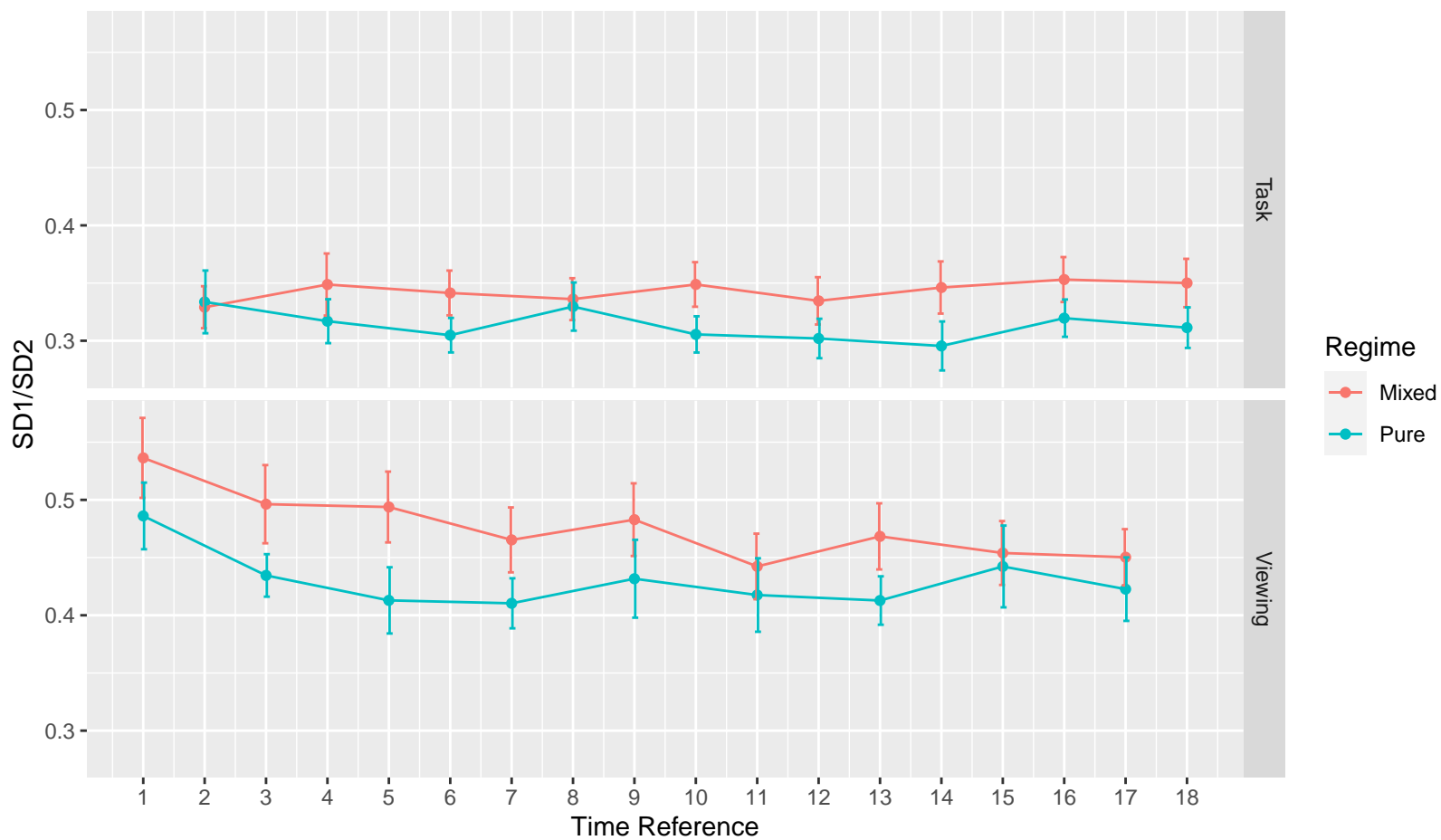

Supplement: Supplementary file 4 — Figure S4. [file PHY2-11-e15863-s001.pdf]

DFA

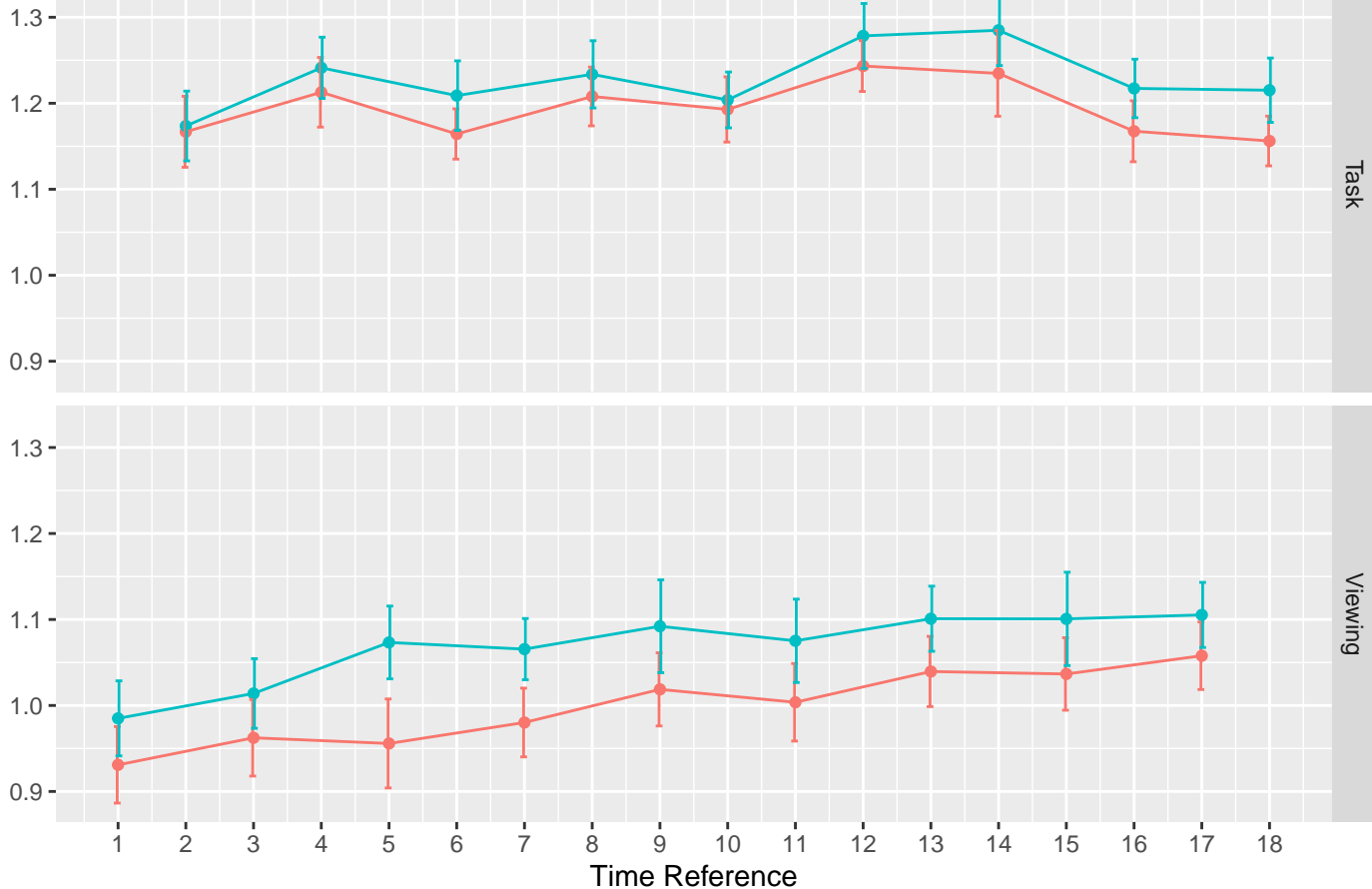

Supplement: Supplementary file 5 — Figure S5. [file PHY2-11-e15863-s005.pdf]

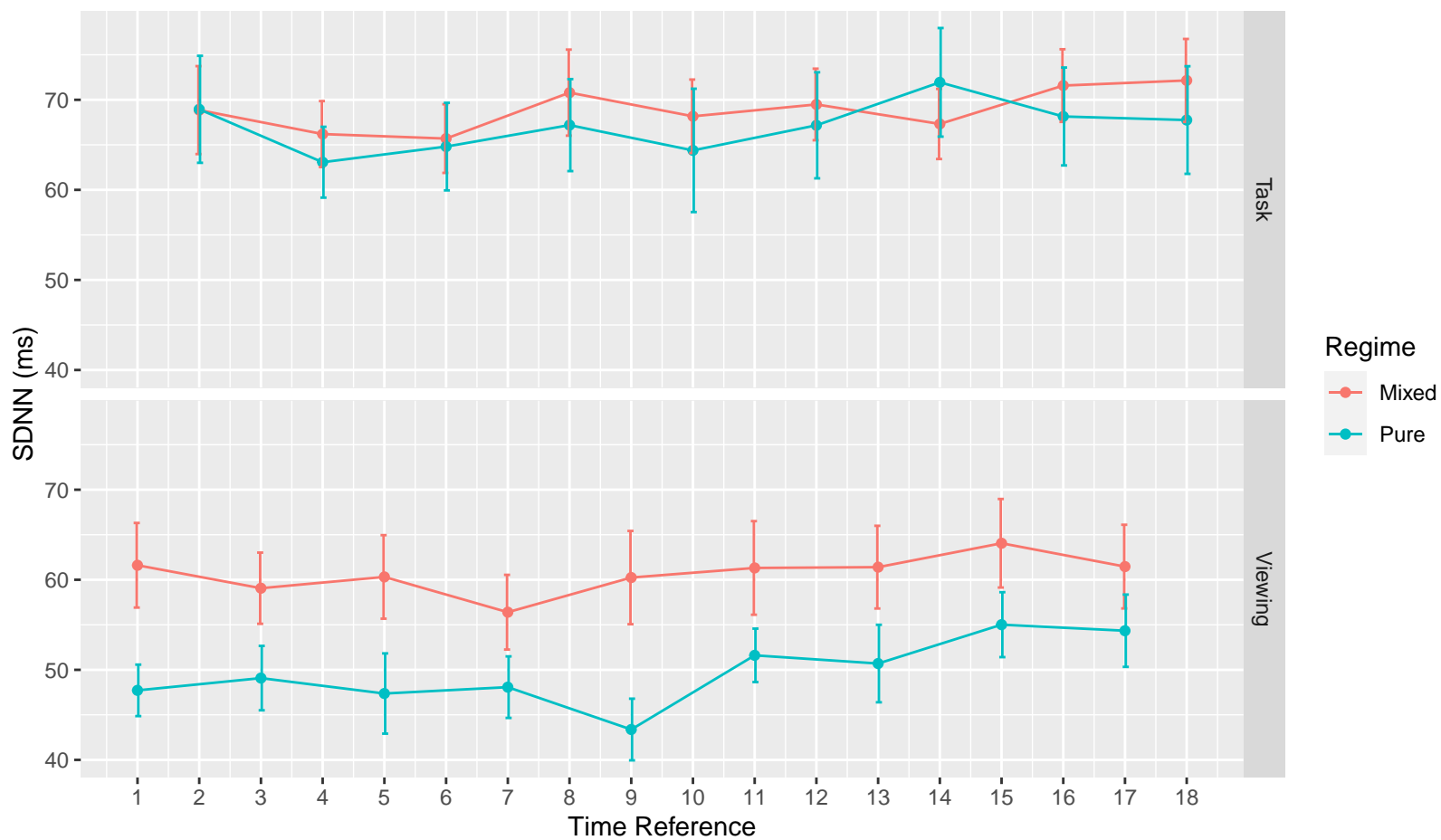

Supplement: Supplementary file 6 — Figure S6. [file PHY2-11-e15863-s004.pdf]
