# Supplementary material for: The effectiveness of time domain and nonlinear heart rate variability metrics in ultra‐short time series
Source: Physiol Rep. 2023 Nov 27;11(22):e15863. doi: 10.14814/phy2.15863 (PMC10681424; doi:10.14814/phy2.15863)
Supplement: Supplementary file 7 — Tables S1‐Table S8. [file PHY2-11-e15863-s003.docx]

Supplemental Table **RMSSD comparison between Pure Regime and Mixed Regime participants in both viewing and task. All numbers except p-values are presented as mean (SD).**

|  | Seg. 1 | Seg. 2 | Seg. 3 | Seg. 4 | Seg. 5 | Seg. 6 | Seg. 7 | Seg. 8 | Seg. 9 |
| --- | --- | --- | --- | --- | --- | --- | --- | --- | --- |
| PURE view | 41.4 (20.2) | 38.5 (19.2) | 35.8 (22.3) | 37.2 (22.3) | 35.1 (23.8) | 38.9 (21.9) | 40.2 (26.8) | 44.7 (28.5) | 43.5 (29.1) |
| MIX view | 59.4 (33.8) | 53.1 (31.8) | 53.6 (32.2) | 48.6 (28.1) | 53.2 (35.7) | 50.7 (37.1) | 52.1 (31.3) | 54.4 (35.9) | 51.2 (29.9) |
| *p* | 0.015 | 0.025 | 0.018 | 0.068 | 0.022 | 0.118 | 0.095 | 0.230 | 0.286 |
| PURE task | 44.2 (31.6) | 37.1 (14.3) | 38.8 (25.0) | 42.8 (30.2) | 38.4 (30.2) | 39.9 (30.8) | 42.5 (33.7) | 42.2 (29.1) | 42.1 (31.2) |
| MIX task | 43.8 (22.3) | 44.6 (25.4) | 43.3 (20.8) | 46.5 (25.4) | 46.1 (25.2) | 44.5 (23.1) | 44.7 (23.3) | 47.9 (21.0) | 48.9 (25.7) |
| *p* | 0.951 | 0.160 | 0.455 | 0.602 | 0.305 | 0.516 | 0.774 | 0.378 | 0.376 |

Supplemental Table **pNN50 comparison between Pure Regime and Mixed Regime participants in both viewing and task. All numbers except p-values are presented as mean (SD).**

|  | Seg. 1 | Seg. 2 | Seg. 3 | Seg. 4 | Seg. 5 | Seg. 6 | Seg. 7 | Seg. 8 | Seg. 9 |
| --- | --- | --- | --- | --- | --- | --- | --- | --- | --- |
| PURE view | 0.20  (0.18) | 0.19  (0.17) | 0.14  (0.16) | 0.15 (0.16) | 0.13 (0.15) | 0.17 (0.15) | 0.15 (0.16) | 0.18 (0.17) | 0.17 (0.16) |
| MIX view | 0.25  (0.23) | 0.26  (0.22) | 0.25  (0.21) | 0.24 (0.21) | 0.24 (0.20) | 0.24 (0.21) | 0.25 (0.21) | 0.25 (0.21) | 0.25 (0.21) |
| *p* | 0.332 | 0.124 | 0.026 | 0.046 | 0.018 | 0.101 | 0.024 | 0.151 | 0.072 |
| PURE task | 0.17  (0.15) | 0.15  (0.11) | 0.14 (0.12) | 0.16 (0.13) | 0.14 (0.14) | 0.15 (0.14) | 0.15 (0.14) | 0.17 (0.15) | 0.18 (0.17) |
| MIX task | 0.18  (0.16) | 0.20  (0.16) | 0.21 (0.17) | 0.20 (0.16) | 0.22 (0.17) | 0.19 (0.15) | 0.21 (0.17) | 0.23 (0.16) | 0.23 (0.17) |
| *p* | 0.807 | 0.142 | 0.111 | 0.223 | 0.048 | 0.263 | 0.172 | 0.117 | 0.271 |

Supplemental Table **SD2 comparison between Pure Regime and Mixed Regime participants in both viewing and task. All numbers except p-values are presented as mean (SD).**

|  | Seg. 1 | Seg. 2 | Seg. 3 | Seg. 4 | Seg. 5 | Seg. 6 | Seg. 7 | Seg. 8 | Seg. 9 |
| --- | --- | --- | --- | --- | --- | --- | --- | --- | --- |
| PURE view | 60.0 (20.3) | 62.8 (25.7) | 61.1 (27.5) | 61.5 (23.1) | 55.2 (19.4) | 66.3 (21.2) | 65.1 (29.6) | 69.5 (22.7) | 69.2 (26.7) |
| MIX view | 75.4 (29.9) | 73.2 (27.0) | 75.2 (31.6) | 71.1 (30.2) | 75.2 (37.2) | 77.3 (35.4) | 77.5 (33.8) | 80.8 (34.6) | 78.1 (34.1) |
| *p* | 0.022 | 0.109 | 0.082 | 0.145 | 0.009 | 0.127 | 0.110 | 0.123 | 0.234 |
| PURE task | 91.7 (39.2) | 84.8 (28.3) | 87.2 (32.8) | 89.5 (35.1) | 86.6 (45.2) | 90.3 (41.2) | 96.3 (40.3) | 91.1 (39.5) | 90.7 (38.8) |
| MIX task | 91.8 (33.8) | 87.3 (26.1) | 87.3 (28.1) | 93.9 (34.7) | 90.2 (29.7) | 92.5 (29.2) | 89.1 (27.4) | 94.9 (28.7) | 95.4 (33.7) |
| *p* | 0.991 | 0.735 | 0.990 | 0.620 | 0.729 | 0.807 | 0.431 | 0.663 | 0.622 |

Supplemental Table **SD1/SD2 comparison between Pure Regime and Mixed Regime participants in both viewing and task. All numbers except p-values are presented as mean (SD).**

|  | Seg. 1 | Seg. 2 | Seg. 3 | Seg. 4 | Seg. 5 | Seg. 6 | Seg. 7 | Seg. 8 | Seg. 9 |
| --- | --- | --- | --- | --- | --- | --- | --- | --- | --- |
| PURE view | 0.49 (0.17) | 0.43 (0.11) | 0.41 (0.14) | 0.41 (0.12) | 0.43 (0.17) | 0.42 (0.18) | 0.41 (0.12) | 0.44 (0.19) | 0.42 (0.16) |
| MIX view | 0.54 (0.19) | 0.50 (0.20) | 0.49 (0.17) | 0.47 (0.17) | 0.48 (0.19) | 0.44 (0.17) | 0.47 (0.17) | 0.45 (0.16) | 0.45 (0.14) |
| *p* | 0.269 | 0.115 | 0.059 | 0.127 | 0.272 | 0.563 | 0.123 | 0.796 | 0.455 |
| PURE task | 0.33 (0.14) | 0.32 (0.10) | 0.30 (0.08) | 0.33 (0.11) | 0.31 (0.08) | 0.30 (0.09) | 0.30 (0.11) | 0.32 (0.09) | 0.31 (0.09) |
| MIX task | 0.33 (0.10) | 0.35 (0.15) | 0.34 (0.11) | 0.34 (0.10) | 0.35 (0.11) | 0.33 (0.12) | 0.35 (0.12) | 0.35 (0.10) | 0.35 (0.12) |
| *p* | 0.888 | 0.339 | 0.141 | 0.816 | 0.088 | 0.227 | 0.108 | 0.191 | 0.162 |

Supplemental Table **DFA alpha1 comparison between Pure Regime and Mixed Regime participants in both viewing and task. All numbers except p-values are presented as mean (SD).**

|  | Seg. 1 | Seg. 2 | Seg. 3 | Seg. 4 | Seg. 5 | Seg. 6 | Seg. 7 | Seg. 8 | Seg. 9 |
| --- | --- | --- | --- | --- | --- | --- | --- | --- | --- |
| PURE view | 0.99  (0.25) | 1.01  (0.23) | 1.07  (0.21) | 1.07  (0.20) | 1.09  (0.27) | 1.08  (0.28) | 1.10  (0.21) | 1.10  (0.30) | 1.11  (0.22) |
| MIX view | 0.93 (0.24) | 0.96  (0.26) | 0.96  (0.29) | 0.98  (0.24) | 1.02  (0.25) | 1.00  (0.26) | 1.04  (0.25) | 1.04  (0.25) | 1.06  (0.23) |
| *p* | 0.389 | 0.395 | 0.085 | 0.116 | 0.291 | 0.283 | 0.275 | 0.355 | 0.387 |
| PURE task | 1.17  (0.21) | 1.24  (0.18) | 1.21  (0.21) | 1.23  (0.21) | 1.20  (0.17) | 1.28  (0.21) | 1.28  (0.22) | 1.22  (0.19) | 1.22  (0.19) |
| MIX task | 1.17  (0.22) | 1.21  (0.23) | 1.16  (0.17) | 1.21  (0.19) | 1.19  (0.22) | 1.24  (0.17) | 1.23  (0.27) | 1.17  (0.19) | 1.16  (0.16) |
| *p* | 0.908 | 0.600 | 0.375 | 0.622 | 0.826 | 0.468 | 0.441 | 0.315 | 0.217 |

Supplemental Table 6). **SDNN comparison between Pure Regime and Mixed Regime participants in both viewing and task. All numbers except p-values are presented as mean (SD).**

|  | Seg. 1 | Seg. 2 | Seg. 3 | Seg. 4 | Seg. 5 | Seg. 6 | Seg. 7 | Seg. 8 | Seg. 9 |
| --- | --- | --- | --- | --- | --- | --- | --- | --- | --- |
| PURE view | 47.7 (16.4) | 49.1 (20.5) | 47.4 (21.8) | 48.1 (19.6) | 43.4 (17.1) | 51.6 (17.0) | 50.7 (24.3) | 55.0 (19.8) | 54.3 (23.1) |
| MIX view | 61.6 (25.7) | 59.1 (23.4) | 60.3 (26.2) | 56.4 (24.5) | 60.2 (30.6) | 61.3 (30.3) | 61.4 (27.6) | 64.1 (29.1) | 61.5 (27.5) |
| *p* | 0.015 | 0.066 | 0.049 | 0.126 | 0.009 | 0.111 | 0.094 | 0.143 | 0.250 |
| PURE task | 68.9 (30.9) | 63.1 (20.4) | 64.8 (25.7) | 67.2 (28.0) | 64.4 (34.9) | 67.2 (32.3) | 71.9 (31.9) | 68.1 (30.7) | 67.8 (31.0) |
| MIX task | 68.9 (25.8) | 66.2 (20.7) | 65.7 (21.6) | 70.8 (27.0) | 68.2 (23.4) | 69.5 (22.5) | 67.3 (21.3) | 71.6 (21.7) | 72.2 (26.1) |
| *p* | 0.991 | 0.563 | 0.886 | 0.608 | 0.637 | 0.746 | 0.523 | 0.614 | 0.563 |

Supplemental Table 7). **Time-domain metrics one-minute comparison during Phase I. p-values are reported in comparison with previous activity. All numbers except p-values are presented as mean (SD).**

|  | SDNN | *p* | RMSSD | *p* | pNN50 | *p* |
| --- | --- | --- | --- | --- | --- | --- |
| Viewing1 min1 | 52.6 (22.2) | - | 47.5 (30.3) | - | 0.23 (0.20) | - |
| Viewing1 min2 | 51.1 (26.0) | 0.572 | 49.1 (32.9) | 0.640 | 0.21 (0.21) | 0.457 |
| Task1 min1 | 46.2 (21.7) | 0.126 | 37.7 (22.7) | 0.005 | 0.18 (0.19) | 0.046 |
| Task1 min2 | 71.8 (33.4) | <0.0014 | 47.3 (33.5) | 0.013 | 0.17 (0.14) | 0.567 |
| Viewing2 min1 | 56.5 (24.0) | <0.0014 | 48.0 (27.5) | 0.803 | 0.25 (0.21) | <0.0014 |
| Viewing2 min2 | 46.8 (23.4) | <0.0014 | 42.6 (29.7) | 0.033 | 0.20 (0.20) | <0.0014 |
| Task2 min1 | 44.2 (22.8) | 0.278 | 37.5 (25.5) | 0.031 | 0.16 (0.19) | <0.0014 |
| Task2 min2 | 68.0 (22.1) | <0.0014 | 42.7 (20.5) | 0.062 | 0.19 (0.13) | 0.237 |
| Viewing3 min1 | 57.5 (28.0) | 0.005 | 47.9 (31.9) | 0.357 | 0.22 (0.20) | 0.034 |
| Viewing3 min2 | 46.6 (24.5) | <0.0014 | 41.7 (31.0) | 0.142 | 0.19 (0.19) | <0.0014 |
| Task3 min1 | 44.5 (21.0) | 0.925 | 36.4 (19.8) | 0.183 | 0.17 (0.18) | 0.116 |
| Task3 min2 | 69.5 (27.9) | <0.0014 | 43.9 (27.6) | 0.012 | 0.19 (0.15) | 0.186 |
| Viewing4 min1 | 53.7 (24.0) | <0.0014 | 43.6 (25.7) | 0.553 | 0.21 (0.20) | 0.235 |
| Viewing4 min2 | 46.4 (23.5) | <0.0014 | 41.4 (28.3) | 0.367 | 0.19 (0.19) | 0.003 |
| Task4 min1 | 46.2 (20.4) | 0.615 | 36.6 (19.9) | 0.061 | 0.16 (0.16) | 0.040 |
| Task4 min2 | 74.2 (33.1) | <0.0014 | 49.8 (34.9) | <0.0014 | 0.20 (0.16) | 0.004 |
| Viewing5 min1 | 56.9 (31.9) | <0.0014 | 48.6 (38.5) | 0.556 | 0.21 (0.19) | 0.613 |
| Viewing5 min2 | 45.4 (23.8) | <0.0014 | 40.8 (27.6) | 0.037 | 0.18 (0.18) | <0.0014 |
| Task5 min1 | 45.8 (23.2) | 0.701 | 38.0 (27.2) | 0.344 | 0.17 (0.17) | 0.174 |
| Task5 min2 | 73.1 (32.8) | <0.0014 | 45.5 (29.7) | 0.011 | 0.19 (0.16) | 0.081 |
| Viewing6 min1 | 62.0 (27.8) | 0.005 | 48.5 (34.8) | 0.483 | 0.22 (0.19) | 0.010 |
| Viewing6 min2 | 46.3 (24.1) | <0.0014 | 40.1 (27.6) | <0.0014 | 0.19 (0.19) | <0.0014 |
| Task6 min1 | 49.3 (23.3) | 0.029 | 37.1 (22.1) | 0.751 | 0.16 (0.16) | 0.541 |
| Task6 min2 | 72.6 (31.4) | <0.0014 | 45.7 (32.0) | <0.0014 | 0.18 (0.15) | 0.217 |
| Viewing7 min1 | 61.6 (30.2) | <0.0014 | 49.5 (32.7) | 0.944 | 0.22 (0.20) | 0.012 |
| Viewing7 min2 | 45.6 (25.0) | <0.0014 | 41.4 (29.7) | 0.009 | 0.19 (0.20) | <0.0014 |
| Task7 min1 | 46.7 (25.0) | 0.279 | 36.6 (23.4) | 0.155 | 0.16 (0.17) | 0.221 |
| Task7 min2 | 77.8 (30.9) | <0.0014 | 48.5 (34.4) | <0.0014 | 0.20 (0.16) | 0.007 |
| Viewing8 min1 | 64.1 (29.9) | <0.0014 | 51.4 (37.5) | 0.361 | 0.23 (0.20) | 0.579 |
| Viewing8 min2 | 50.9 (25.6) | <0.0014 | 45.9 (31.6) | 0.147 | 0.21 (0.20) | 0.090 |
| Task8 min1 | 50.7 (23.7) | 0.941 | 40.3 (25.1) | 0.070 | 0.19 (0.19) | 0.029 |
| Task8 min2 | 76.5 (30.3) | <0.0014 | 47.5 (27.9) | 0.005 | 0.20 (0.14) | 0.263 |
| Viewing9 min1 | 61.8 (30.1) | <0.0014 | 50.6 (34.2) | 0.518 | 0.22 (0.19) | 0.059 |
| Viewing9 min2 | 49.0 (23.6) | <0.0014 | 42.4 (27.4) | 0.010 | 0.21 (0.21) | 0.456 |
| Task9 min1 | 51.6 (24.5) | 0.832 | 43.1 (27.4) | 0.422 | 0.21 (0.19) | 0.054 |
| Task9 min2 | 75.0 (32.6) | <0.0014 | 46.9 (30.6) | 0.109 | 0.20 (0.17) | 0.893 |

Supplemental Table 8). **Nonlinear metrics one-minute comparison during Phase I. p-values are reported in comparison with previous activity. All numbers except p-values are presented as mean (SD).**

|  | SD1/SD2 | *p* | SD2 | *p* | DFA | *p* |
| --- | --- | --- | --- | --- | --- | --- |
| Viewing1 min1 | 0.51 (0.20) | - | 65.1 (25.6) | - | 0.99 (0.29) | - |
| Viewing1 min2 | 0.54 (0.19) | 0.117 | 62.6 (30.4) | 0.355 | 0.91 (0.27) | 0.008 |
| Task1 min1 | 0.45 (0.16) | <0.0014 | 58.6 (27.3) | 0.314 | 1.01 (0.24) | 0.034 |
| Task1 min2 | 0.34 (0.14) | <0.0014 | 95.0 (42.2) | <0.0014 | 1.25 (0.22) | <0.0014 |
| Viewing2 min1 | 0.48 (0.17) | <0.0014 | 70.5 (28.8) | <0.0014 | 1.00 (0.26) | <0.0014 |
| Viewing2 min2 | 0.51 (0.20) | 0.051 | 57.9 (27.6) | <0.0014 | 0.97 (0.26) | 0.285 |
| Task2 min1 | 0.48 (0.19) | 0.230 | 55.6 (28.6) | 0.410 | 1.11 (0.27) | <0.0014 |
| Task2 min2 | 0.33 (0.10) | <0.0014 | 90.7 (29.3) | <0.0014 | 1.25 (0.20) | <0.0014 |
| Viewing3 min1 | 0.47 (0.17) | <0.0014 | 72.4 (34.3) | <0.0014 | 1.04 (0.29) | <0.0014 |
| Viewing3 min2 | 0.50 (0.18) | 0.235 | 58.0 (28.6) | <0.0014 | 0.97 (0.30) | 0.053 |
| Task3 min1 | 0.46 (0.15) | 0.015 | 56.7 (27.1) | 0.731 | 1.07 (0.28) | 0.002 |
| Task3 min2 | 0.32 (0.10) | <0.0014 | 92.7 (35.5) | <0.0014 | 1.22 (0.20) | <0.0014 |
| Viewing4 min1 | 0.44 (0.15) | <0.0014 | 67.9 (29.3) | <0.0014 | 1.02 (0.26) | <0.0014 |
| Viewing4 min2 | 0.50 (0.21) | 0.007 | 57.8 (28.4) | <0.0014 | 1.01 (0.27) | 0.639 |
| Task4 min1 | 0.45 (0.17) | 0.097 | 59.1 (26.6) | 0.940 | 1.15 (0.32) | <0.0014 |
| Task4 min2 | 0.34 (0.12) | <0.0014 | 98.0 (41.5) | <0.0014 | 1.22 (0.22) | 0.085 |
| Viewing5 min1 | 0.46 (0.18) | <0.0014 | 71.3 (37.8) | <0.0014 | 1.06 (0.29) | <0.0014 |
| Viewing5 min2 | 0.50 (0.21) | 0.067 | 56.3 (28.9) | <0.0014 | 1.02 (0.28) | 0.337 |
| Task5 min1 | 0.46 (0.18) | 0.008 | 57.9 (28.3) | 0.414 | 1.08 (0.24) | 0.1 |
| Task5 min2 | 0.32 (0.09) | <0.0014 | 97.8 (42.2) | <0.0014 | 1.24 (0.22) | <0.0014 |
| Viewing6 min1 | 0.43 (0.20) | <0.0014 | 78.8 (33.8) | <0.0014 | 1.05 (0.32) | <0.0014 |
| Viewing6 min2 | 0.49 (0.20) | 0.003 | 57.6 (28.6) | <0.0014 | 1.00 (0.27) | 0.174 |
| Task6 min1 | 0.42 (0.16) | <0.0014 | 63.7 (30.4) | 0.010 | 1.18 (0.26) | <0.0014 |
| Task6 min2 | 0.32 (0.10) | <0.0014 | 97.0 (39.8) | <0.0014 | 1.27 (0.20) | 0.004 |
| Viewing7 min1 | 0.44 (0.16) | <0.0014 | 78.3 (37.5) | <0.0014 | 1.09 (0.28) | <0.0014 |
| Viewing7 min2 | 0.50 (0.18) | 0.013 | 56.6 (29.7) | <0.0014 | 1.02 (0.28) | 0.058 |
| Task7 min1 | 0.44 (0.16) | 0.003 | 59.8 (32.4) | 0.140 | 1.14 (0.28) | 0.003 |
| Task7 min2 | 0.32 (0.13) | <0.0014 | 103.4 (38.6) | <0.0014 | 1.26 (0.23) | <0.0014 |
| Viewing8 min1 | 0.43 (0.17) | <0.0014 | 81.1 (35.4) | <0.0014 | 1.09 (0.28) | <0.0014 |
| Viewing8 min2 | 0.50 (0.21) | 0.002 | 63.3 (30.5) | <0.0014 | 1.03 (0.28) | 0.048 |
| Task8 min1 | 0.45 (0.18) | 0.034 | 64.7 (30.3) | 0.722 | 1.12 (0.29) | 0.02 |
| Task8 min2 | 0.32 (0.09) | <0.0014 | 102.4 (39.7) | <0.0014 | 1.22 (0.19) | 0.002 |
| Viewing9 min1 | 0.44 (0.15) | <0.0014 | 78.2 (36.3) | <0.0014 | 1.11 (0.25) | 0.002 |
| Viewing9 min2 | 0.49 (0.20) | 0.079 | 61.4 (29.2) | <0.0014 | 1.04 (0.28) | 0.035 |
| Task9 min1 | 0.46 (0.18) | 0.135 | 65.2 (30.4) | 0.708 | 1.11 (0.29) | 0.007 |
| Task9 min2 | 0.32 (0.09) | <0.0014 | 100.2 (41.8) | <0.0014 | 1.21 (0.20) | 0.007 |
